# Suppression of FOXO1 attenuates inflamm‐aging and improves liver function during aging

**DOI:** 10.1111/acel.13968

**Published:** 2023-08-21

**Authors:** Wanbao Yang, Da Mi Kim, Wen Jiang, Weiqi Ai, Quan Pan, Shahina Rahman, James J. Cai, Wesley A. Brashear, Yuxiang Sun, Shaodong Guo

**Affiliations:** ^1^ Department of Nutrition, College of Agriculture and Life Sciences Texas A&M University College Station Texas USA; ^2^ Department of Statistics Texas A&M University College Station Texas USA; ^3^ Department of Veterinary Integrative Biosciences Texas A&M University College Station Texas USA; ^4^ High Performance Research Computing Texas A&M University College Station Texas USA

**Keywords:** aging, FOXO1, inflamm‐aging, inflammation, Kupffer cell, liver function, monocyte‐derived macrophage

## Abstract

The liver is a key metabolic organ that maintains whole‐body nutrient homeostasis. Aging‐induced liver function alterations contribute to systemic susceptibility to aging‐related diseases. However, the molecular mechanisms of liver aging remain insufficiently understood. In this study, we performed bulk RNA‐Seq and single‐cell RNA‐Seq analyses to investigate the underlying mechanisms of the aging‐induced liver function changes. We found that liver inflammation, glucose intolerance, and liver fat deposition were aggravated in old mice. Aging significantly increased pro‐inflammation in hepatic macrophages. Furthermore, we found that Kupffer cells (KCs) were the major driver to induce pro‐inflammation in hepatic macrophages during aging. In KCs, aging significantly increased pro‐inflammatory levels; in monocyte‐derived macrophages (MDMs), aging had a limited effect on pro‐inflammation but led to a functional quiescence in antigen presentation and phagosome process. In addition, we identified an aging‐responsive KC‐specific (ARKC) gene set that potentially mediates aging‐induced pro‐inflammation in KCs. Interestingly, FOXO1 activity was significantly increased in the liver of old mice. FOXO1 inhibition by AS1842856 significantly alleviated glucose intolerance, hepatic steatosis, and systemic inflammation in old mice. FOXO1 inhibition significantly attenuated aging‐induced pro‐inflammation in KCs partially through downregulation of ARKC genes. However, FOXO1 inhibition had a limited effect on aging‐induced functional quiescence in MDMs. These results indicate that aging induces pro‐inflammation in liver mainly through targeting KCs and FOXO1 is a key player in aging‐induced pro‐inflammation in KCs. Thus, FOXO1 could be a potential therapeutic target for the treatment of age‐associated chronic diseases.

## Graphical abstract

Aging activates hepatic FOXO signaling pathway, increases hepatic fat deposition, induces insulin resistance, stimulates pro‐inflammation in Kupffer cells (KCs), and leads to a functional quiescence of monocyte‐infiltrated macrophages (MDMs) in liver. Inhibition of FOXO1 decreases hepatic fat accumulation, improves insulin sensitivity, and attenuates pro‐inflammation in KCs during aging.

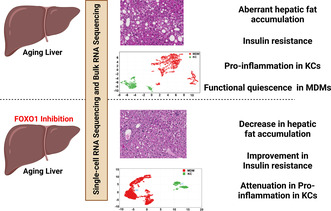

## 1 INTRODUCTION

Aging is a process characterized by the progressive loss of physiological integrity, which increases vulnerability to death. With aging, most individuals develop a chronic, sterile, low‐grade inflammation, called inflamm‐aging (Franceschi et al., 2018). Inflamm‐aging contributes to the pathogenesis of age‐related diseases, including cardiovascular disease, chronic kidney disease, diabetes, cancer, depression, and sarcopenia (Costantino et al., 2016; Ferrucci & Fabbri, 2018; Prattichizzo et al., 2018). Therefore, inflamm‐aging modulation is a potential therapeutic strategy to prevent aging‐related diseases.

Several factors contribute to the development of inflamm‐aging, including genetic susceptibility, visceral obesity, microbiota and gut permeability, cellular senescence, NLRP3 inflammasome activation, oxidative stress caused by mitochondrial dysfunction, immune cells dysregulation, and chronic infection (Ferrucci & Fabbri, 2018). The immune system becomes gradually dysregulated during aging, leading to elevated blood levels of pro‐inflammatory mediators, such as TNFα, IL6, and C‐reactive protein (Harris et al., 1999; Mooradian et al., 1991). Energy homeostasis also becomes dysregulated with aging, which results in the redistribution of subcutaneous fat to visceral regions and contributes to inflammation (Bouchard et al., 1993; Chumlea et al., 1989; Curtis et al., 2005). Metabolism‐induced inflammation, also known as metaflammation, shares similarities with inflamm‐aging, including the elevation of certain circulating pro‐inflammatory cytokines (Prattichizzo et al., 2018). Therefore, the molecules that play a key role in the regulation of metabolic homeostasis potentially mediate the development of chronic inflammation during aging.

Forkhead box O1 (FOXO1) transcription factor has been indicated to be involved in the regulation of nutrient metabolism and energy homeostasis (Cheng et al., 2009; InSug et al., 2015; Matsumoto et al., 2007; Yang et al., 2019; Zhang et al., 2012). Deletion of hepatic *Foxo1* improves glucose homeostasis in insulin resistant mice (Dong et al., 2008). FOXO1 inhibition by AS1842856 attenuates hepatic steatosis in diet‐induced obesity mice (Ding et al., 2020). In mature macrophages, FOXO1 promotes inflammation through the activation of TLR4‐ and STAT6‐mediated signaling pathways (Fan et al., 2010; Lee et al., 2022). In invertebrates, DAF‐16, the *Foxo* homolog gene, mediates the effect of insulin/IGF signaling on lifespan (Ogg et al., 1997). Overexpression of FOXO in *Drosophila* and *C.elegans* increases their lifespan (Giannakou et al., 2004; Henderson & Johnson, 2001). However, studies in mammalians show that FOXO1 does not have a significant correlation with longevity (Chiba et al., 2009; Kleindorp et al., 2011). Considering the role of FOXO1 in regulating glucose metabolism and inflammation, we hypothesize that FOXO1 plays an important role in the regulation of aging‐induced inflammation and dysregulation of glucose homeostasis.

Liver is an important metabolic organ that plays a key role in maintaining whole‐body nutrient homeostasis by regulating energy metabolism, clearing xenobiotic and endobiotic, and synthesizing necessary molecules (Rui, 2014). As a result, aging‐induced changes in liver contribute to systemic susceptibility to aging‐related diseases. Different types of liver cells, including hepatocytes, endothelial cells, hepatic stellate cells (HSC), and macrophages, are all affected by the aging process (Hunt et al., 2019). However, most studies on liver aging focused on whole‐liver tissue, which is mainly composed of parenchymal cells, hepatocytes. Thus, the effects of aging on liver nonparenchymal cells (NPCs) are less understood. In this study, we used bulk RNA‐Seq and single‐cell RNA (scRNA)‐Seq technologies to analyze aging‐induced changes, and the role of FOXO1 in aging‐related processes in both whole‐liver and individual liver cells, particularly liver macrophages. We found that insulin resistance, liver fat accumulation, liver inflammation, and systemic inflammation were significantly aggravated in old mice. Additionally, aging significantly increased pro‐inflammatory response in Kupffer cells (KCs) and induced a functional quiescence in monocyte‐derived macrophages (MDMs). FOXO1 activity was significantly enhanced in the livers of old mice and FOXO1 inhibition improved insulin resistance, hepatic steatosis, and inflammation in old mice. Furthermore, we found that FOXO1 inhibition attenuated aging‐induced pro‐inflammation in KCs and had a limited effect on aging‐induced functional quiescence in MDMs. Taken together, this study indicates that FOXO1 plays an important role in the liver aging processes and suggests that FOXO1 is a potential therapeutic target for the treatment of aging‐induced chronic diseases.

## 2 RESULTS

### 2.1 Lipid and glucose homeostasis are impaired in old mice

**FIGURE 1:**
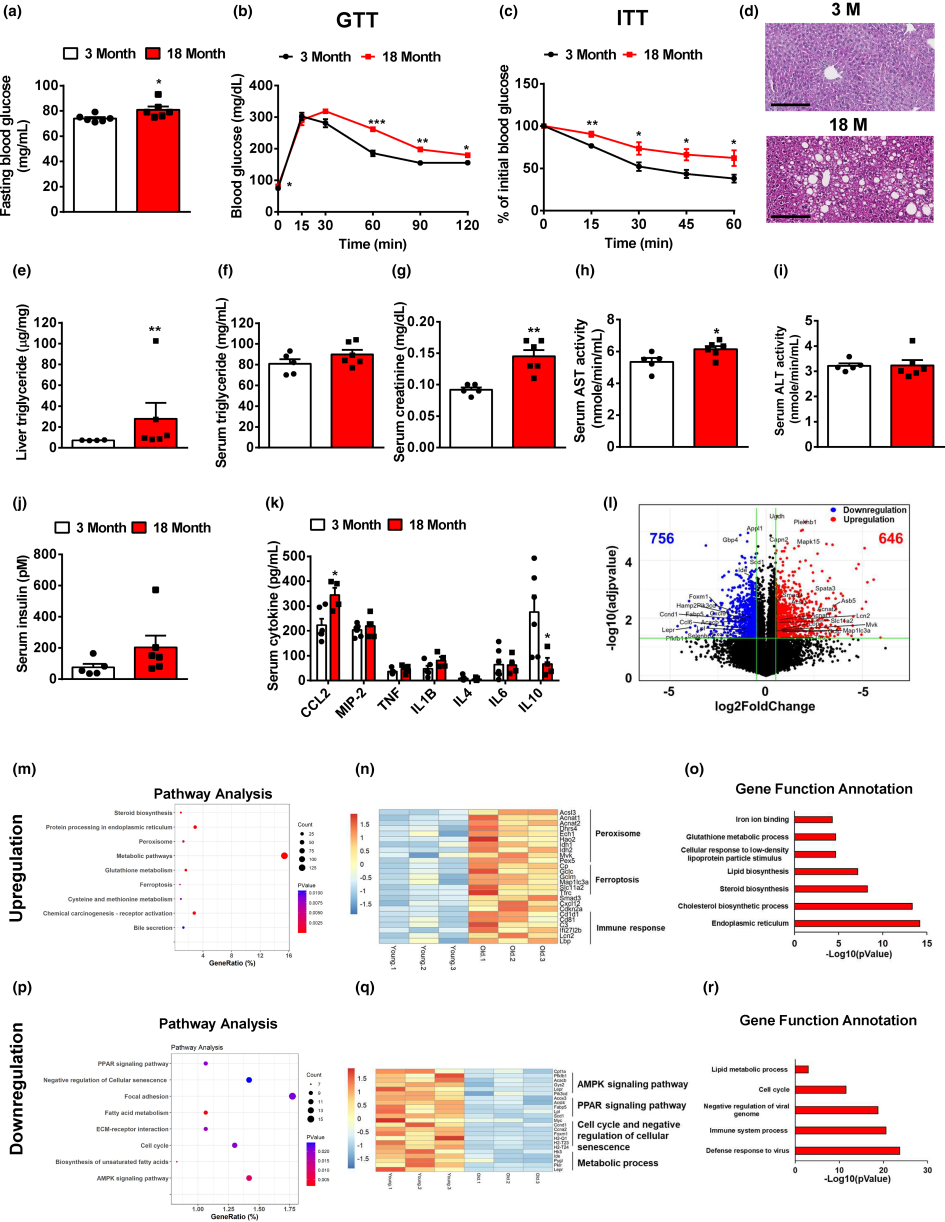
Lipid and glucose homeostasis are impaired in old mice. (a) Overnight (16 h) fasting blood glucose levels in young and old mice, *n* = 6 mice/group. (b) Glucose tolerance tests in young and old mice, *n* = 6 mice/group. (c) Insulin tolerance tests in young and old mice, *n* = 6 mice/group. (d) H&E staining of the livers of young and old mice. Representative images were shown. (e) Liver triglycerides in young and old mice, *n* = 4–6 mice/group. (f–j) Serum triglyceride (f), creatinine (g), AST (h), ALT (i), and insulin (j) in young and old mice, *n* = 5 mice/group. (k) Serum cytokine levels in young and old mice, *n* = 5–8 mice/group. (l) Volcano plot of hepatic gene expression in young and old mice analyzed by RNA‐Seq of total liver mRNA. (m) Pathway analysis of upregulated genes in the livers of old mice. (n) Heatmap of representative genes upregulated in the livers of old mice. (o) Gene function annotation of upregulated genes in the livers of old mice. (p) Pathway analysis of downregulated genes in the livers of old mice. (q) Heatmap of representative genes downregulated in the livers of old mice. (r) Gene function annotation of downregulated genes in the livers of old mice. All data are presented as mean ± SEM. *p < 0.05, **p < 0.01, ***p < 0.001.

In old (18‐month‐old) mice, overnight fasting blood glucose was significantly increased by 10% compared to that in young (3‐month‐old) mice (Figure 1a). The old mice showed a significant glucose intolerance and insulin resistance, as compared to young mice (Figure 1b,c). Additionally, liver fat deposition and triglyceride levels were markedly increased in old mice (Figure 1d,e; Figure S1A). The old mice had a nonsignificant increase in serum triglyceride, significant increases in serum creatinine and AST (58% and 15%, respectively), and no significant changes in serum ALT (Figure 1f–i). As compared to young mice, serum cholesterol and non‐esterified fatty acids (NEFA) levels were significantly decreased by 14% and 21% in old mice, respectively, without significant changes in serum LDL and HDL (Figure S1B–E). The old mice showed a nonsignificant increase in serum insulin by 1.7‐fold as compared to young mice (Figure 1j). Serum cytokine profile analysis showed that compared to young mice, the old mice had an increase in serum chemokine and pro‐inflammatory cytokines, as indicated by a significant increase in serum CCL2 by 54% and an increasing trend in serum TNF and IL1B. Serum anti‐inflammatory cytokine such as IL10 was significantly decreased by 77% in old mice (Figure 1k). To investigate the underlying mechanisms of liver aging, we performed RNA‐Seq analysis in the livers of young and old mice. Compared to young mice, the old mice showed 646 and 756 differentially expressed genes with more than 1.5‐fold increase and decrease in mRNA expression, respectively (Figure 1l). Pathway and gene function analyses showed that the upregulated gene set was involved in the regulation of peroxisome, ferroptosis, and lipid biosynthesis. Of note, the expression levels of genes responsible for immune response were upregulated (Figure 1m–o). On the other hand, the downregulated gene set was involved in AMPK, PPAR, and glucose metabolism signaling pathways. The expression levels of genes responsible for cell cycle and negative regulation of cellular senescence were downregulated. Gene function analysis showed that immune defense response was significantly attenuated in the old mouse livers (Figure 1p–r). Taken together, these results suggest that glucose tolerance, lipid homeostasis, and immune response are significantly impaired in old mice.

### 2.2 Aging enhances pro‐inflammation in hepatic macrophages

**FIGURE 2:**
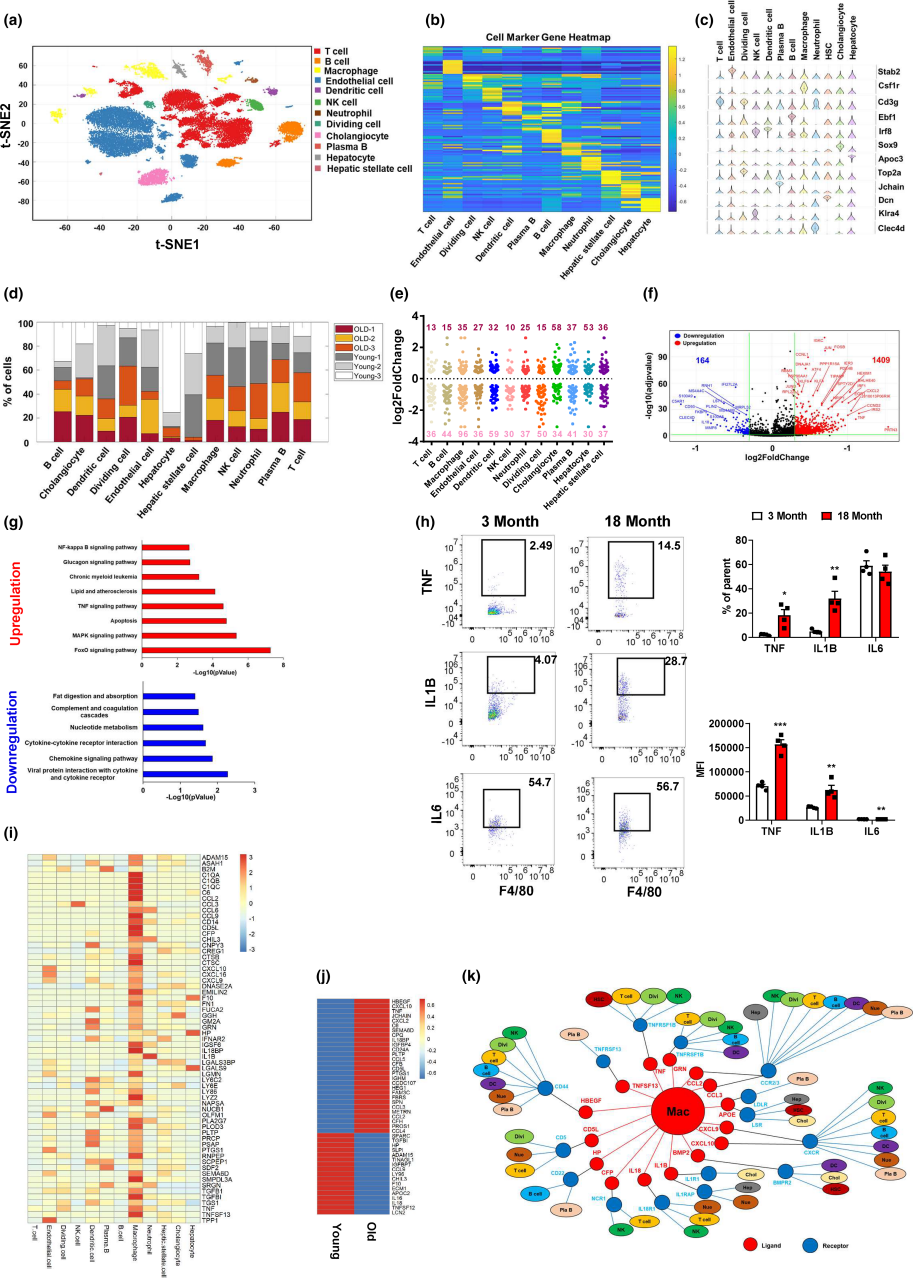
Aging enhances pro‐inflammation in hepatic macrophages. (a) t‐SNE visualization of liver cell clusters. (b) Heatmap of cluster marker genes. (c) Violin plots of representative marker gene expression for each cluster. (d) Percent contribution of young and old mouse liver cells in each cluster. (e) Cell‐type distribution for differentially expressed genes in the old mouse livers. (f) Volcano plot of gene expression in the hepatic macrophage of young and old mice. (g) Pathway analysis of differentially expressed genes between young and old hepatic macrophages. Red: upregulated genes in old hepatic macrophages; Blue: downregulated genes in old hepatic macrophages. (h) The intracellular intensities of pro‐inflammatory cytokines in the hepatic macrophages of young and old mice. (i) Heatmap of hepatic macrophage specifically secreted factors. (j) Heatmap of aging‐regulated secreted factors that show high expression in hepatic macrophages. (k) Cell–cell communication network analysis between hepatic macrophages and other liver cells.

To elucidate liver cell heterogeneity and their dynamic changes during aging, we performed scRNA‐Seq in the liver NPCs of young and old mice. We obtained a total of 33,412 single‐cell transcriptomes (Young mice: 16,579 and Old mice: 16,833). T‐distributed stochastic neighbor embedding (t‐SNE) visualization of the combined young and old data revealed 12 different cell types, including T cells, B cells, macrophages, endothelial cells, dendritic cells, natural killer (NK) cells, neutrophils, dividing cells, cholangiocytes, plasma B cells, hepatocytes, and HSC, based on marker gene expression (Figure 2a–c). All 12 cell types were derived from both young and old mouse livers. We observed that the old liver accounted for more than 50% of the cells in the dividing cell, macrophage, plasma B, and T‐cell clusters (Figure 2d). The genes most highly expressed in macrophage, dendritic cell, cholangiocyte, or hepatocyte were primarily aging‐regulated genes in liver (Figure 2e).

Hepatic macrophages are the major driver for chronic inflammation in livers. Thus, we analyzed the gene expression profiles in the hepatic macrophages of young and old mice. We identified 1409 upregulated and 164 downregulated genes in the old mouse liver macrophages compared to young mouse macrophages (Figure 2f). The upregulated genes were involved in pro‐inflammatory signaling pathways, including MAPK, TNF, and NF‐kappa B signaling pathways. Of note, the FoxO signaling pathway was significantly enhanced in the old mouse liver macrophages (Figure 2g). On the other hand, the genes involved in cytokine‐receptor interaction and chemokine signaling pathway were significantly downregulated in the hepatic macrophages of the old mice (Figure 2g). We further performed flow cytometry analysis to detect the intracellular pro‐inflammatory cytokine levels in hepatic macrophages as described in Figure S2A,B. Compared to young mice, the percentage as well as median fluorescence intensity (MFI) of intracellular pro‐inflammatory cytokines such as TNF and IL1B were significantly increased in the hepatic macrophages of old mice. Aging had a limited effect on percentage of IL6 but significantly increased MFI of IL6 in the old mouse hepatic macrophages (Figure 2h). Intercellular crosstalk through ligand and receptor signaling plays an important role in organ biology. We further analyzed the macrophage‐specific secretum profile based on the mouse secretum gene list (Xiong et al., 2019). We identified 63 macrophage‐specific ligands, including TNF, IL1B, CCL2, CXCL9, CXCL10, and CXCL10 (Figure 2i). In the hepatic macrophages of old mice, 28 macrophage‐specific ligands were upregulated, including TNF, CCL2, CXCL2, and CXCL10 that are involved in the regulation of pro‐inflammation. There were 17 macrophage‐specific ligands downregulated in liver macrophages during aging, including HP, CHIL3, and IL18 that play an important role in the response of anti‐inflammation (Figure 2j). The ligands secreted by hepatic macrophages regulate the biological functions in other liver cells through a paracrine mechanism. We next constructed the cell–cell communication network between liver macrophages and other liver cells by scTenifoldXct, a semi‐supervised method for predicting cell–cell interactions (Yang, Li, et al., 2023. We found that the CCL2/3‐CCR2/3 and CXCL9/10‐CXCR signaling pathways played a key role in mediating the crosstalk between macrophages and other immune cells in liver. CCL2/3‐CCR2/3, TNF‐TNFRSF1B, IL1B‐IL1RAP, and APOE‐LSR signaling pathways mediated the communication between macrophages and hepatocytes in liver (Figure 2k). Collectively, these results suggest that aging enhances pro‐inflammation in hepatic macrophages, thereby promoting liver inflamm‐aging.

### 2.3 Kupffer cells contribute to the pro‐inflammation in hepatic macrophages during aging

**FIGURE 3:**
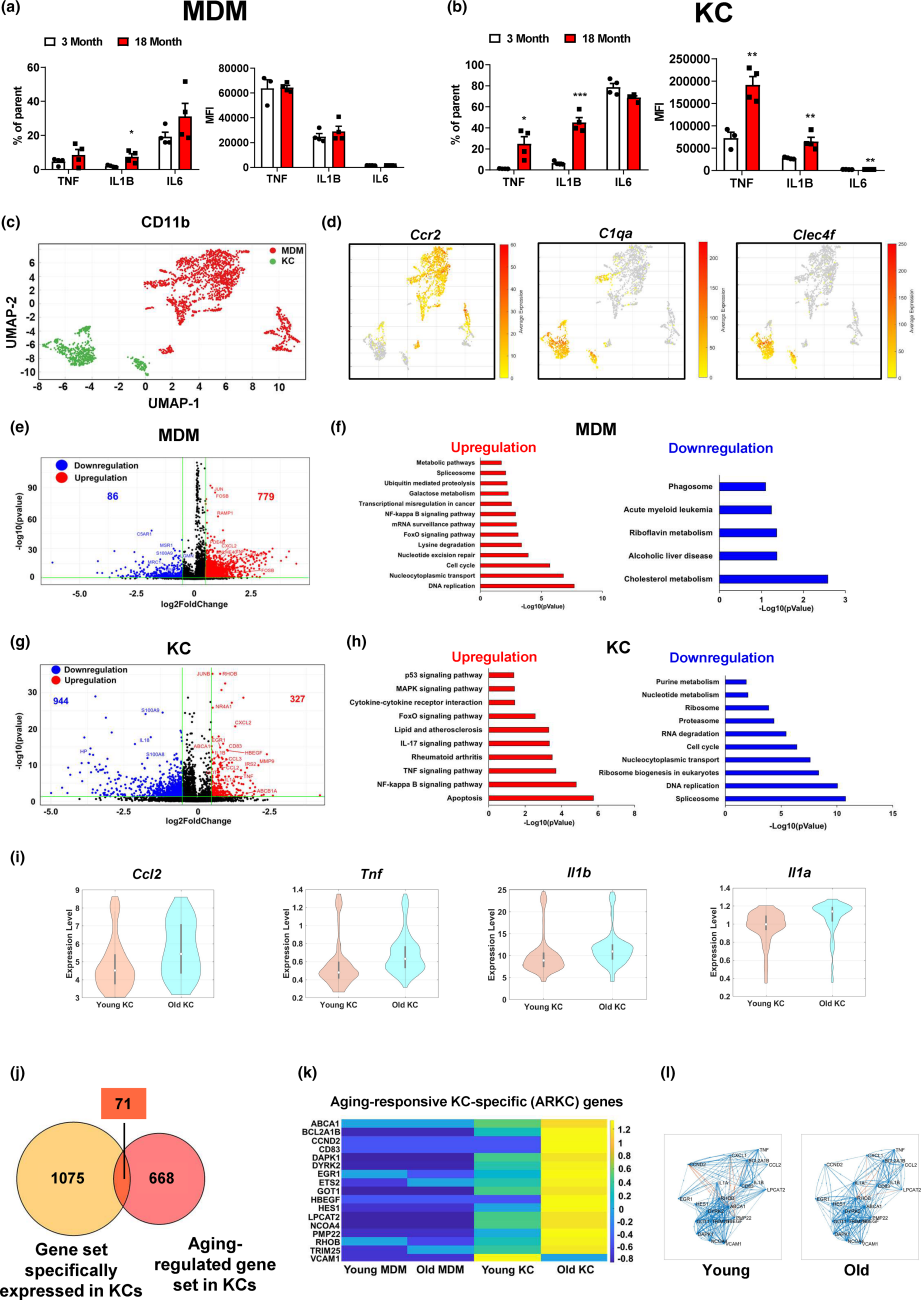
Kupffer cells contribute to the pro‐inflammation in hepatic macrophages during aging. (a, b) The intracellular intensities of pro‐inflammatory cytokines in the MDMs and KCs of young and old mice, *n* = 4 mice/group. (c) Illustration of liver‐resident Kupffer cell (KC, green) and monocyte‐derived macrophage (MDM, red). (d) Feature plot of KC and MDM marker gene expression. (e) Volcano plot of gene expression in the MDMs of young and old mice. (f) Pathway analysis of differentially expressed genes between young and old mouse MDMs. Red: upregulated genes in old MDMs; Blue: downregulated genes in old MDMs. (g) Volcano plot of gene expression in the KCs of young and old mice. (h) Pathway analysis of differentially expressed genes between young and old mouse KCs. Red: upregulated genes in old MDMs; Blue: downregulated genes in old MDMs. (i) Violin plots of pro‐inflammatory cytokine genes in the KCs of young and old mice. (j) Identification of aging‐responsive KC‐specific (ARKC) genes by merging the KC‐specific gene set and aging‐regulated KC gene set. (k) Heatmap of ARKC genes in KCs and MDMs of young and old mice. (l) ARKC gene network analysis in both young and old mice. All data are presented as mean ± SEM. *p < 0.05, **p < 0.01. ***p < 0.001.

To further detect the effect of aging on macrophage features, we separated macrophages into KCs and MDMs based on the intensity of F4/80 (Figure S2B). In MDMs, aging significantly increased the percentage of IL1B but had no significant effect on the percentage and MFI of TNF and IL6 (Figure 3a). In KCs, compared to young mice the percentage and MFI of TNF and IL1B were significantly increased in old mice. The MFI but not percentage of IL6 was significantly increased in the KCs of old mice (Figure 3b). To detect the effect of aging on MDMs and KCs at transcriptomic level, we further analyzed scRNA‐Seq data and divided the macrophage cluster into MDMs and KCs based on their marker gene expression patterns (Figure 3c). MDMs were characterized by a high expression level of the *Ccr2* gene, and KCs showed high expression levels of *Clec4f* and *C1qa* genes (Figure 3d). MDMs and KCs exhibited different secretum profiles: MDMs showed specific expression of *Fn1*, *Hp*, and *Tgfb1*, whereas KCs showed specific expression of *Ccl2*, *Ccl3*, *Ccl6*, and *Il1b* (Figure S3A). The old mouse livers accounted for more than 60% of KCs, whereas young and old mice showed a comparable MDM proportion in liver (Figure S3B). We then analyzed the differentially expressed genes in old vs young MDMs, and identified 779 upregulated and 86 downregulated genes (Figure 3e). Pathway analysis revealed that the upregulated gene set in MDMs is mainly involved in DNA replication, cell cycle, and mRNA process. Of note, the FoxO signaling pathway was enhanced in old MDMs. The downregulated gene set in MDMs was enriched in cholesterol metabolism and phagosome, which indicates that aging potentially impairs the scavenger function in MDMs (Figure 3f). To further determine how aging affects the functional properties of MDMs, we evaluated their M1 polarization based on the expression of signature genes at the single‐cell level (Li et al., 2019). A more pro‐inflammatory phenotype is indicated by a higher macrophage polarization index (MPI). We found that MDMs from the old mouse livers exhibited a notable shift toward less pro‐inflammatory phenotype (Figure S3C). In KCs, aging‐induced 327 upregulated and 944 downregulated genes (Figure 3g). The pathway analysis revealed that the upregulated gene set in old KCs was involved in pro‐inflammation, as indicated by significant enrichments in NF‐kappa B, TNF, IL‐17, and MAPK signaling pathways. Apoptosis and FoxO signaling pathway were significantly enhanced in old KCs (Figure 3h). The downregulated gene set in old KCs was clustered into RNA process, DNA replication, cell cycle, and nucleotide metabolism (Figure 3h). The expression levels of pro‐inflammatory cytokine genes, including *Ccl2*, *Tnf*, *Il1b*, and *Il1a*, were significantly increased in old KCs (Figure 3i). MPI analysis showed that the KCs from old mouse liver exhibited a slight shift toward a pro‐inflammatory phenotype (Figure S3D). Considering that KCs play a major role in the aging‐induced pro‐inflammation in hepatic macrophages, we further investigated the underlying mechanisms of aging‐induced pro‐inflammation in KCs by analyzing the genes specifically expressed in KCs and significantly regulated by aging in KCs; this gene set potentially plays a key role in regulating aging‐induced pro‐inflammation in KCs. We found 71 genes by merging KC‐specific gene set and aging‐regulated gene set in KCs (Figure 3j). To focus on the genes with high expression levels in KC, we excluded the genes with average expression of less than 0.8. We then screened out 17 aging‐responsive and KC‐specific (ARKC) genes (Figure 3k; Figure S3E). Network analysis of these 17 genes indicated that aging markedly altered crosstalk between these 17 genes and pro‐inflammatory cytokine genes including *Ccl2*, *Tnf*, *Il1b*, and *Il1a* (Figure 3l). Taken together, these results indicate that KCs contribute to the aging‐induced pro‐inflammation in hepatic macrophages.

### 2.4 KCs and MDMs are differentially regulated by aging in liver

**FIGURE 4:**
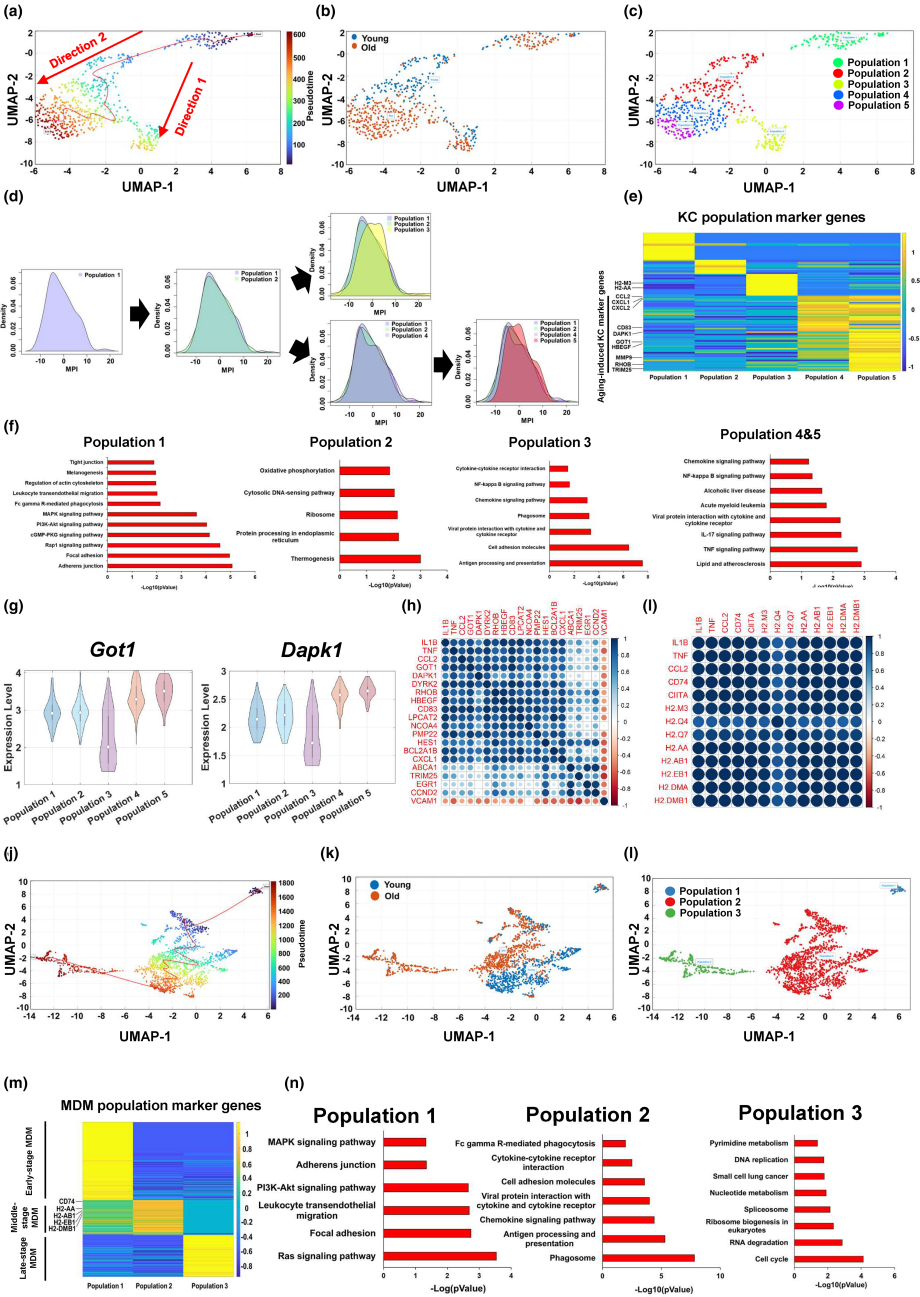
KCs and MDMs are differentially affected during aging. (a) Pseudotime analysis of the KCs from young and old mice. (b) Illustration of the KCs from young and old mice. (c) The KCs were divided into five populations based on their pseudotime analysis. (d) Macrophage polarization index (MPI) analysis in different KC populations. (e) Heatmap of KC population marker genes. (f) Pathway analysis of KC population marker genes. (g) Violin plots of ARKC genes *Got1* and *Dapk1* in KC populations. (h) Correlation between ARKC genes and pro‐inflammatory cytokine genes in KC populations 4 and 5. (i) Correlation between antigen presentation/process genes and pro‐inflammatory cytokine genes in KC population 3. (j) Pseudotime analysis of the MDMs from young and old mice. (k) Illustration of the MDMs from young and old mice. (l) The MDMs were divided into three populations based on their pseudotime analysis. (m) Heatmap of MDM population marker genes. (n) Pathway analysis of MDM population marker genes.

To further investigate the aging process of hepatic macrophages, we performed pseudotime analysis and found that there appeared to be two different directions in KCs during aging (Figure 4a). The terminus of KC direction 1 contained both young and old KCs, whereas the terminus of KC direction 2 presented KCs only from the old mouse liver (Figure 4b). We then divided KCs into five different populations based on their pseudotime, including populations 1 and 2 (early stage), population 3 (direction 1), populations 4 and 5 (late stage, direction 2; Figure 4c). We then performed MPI analysis and found that populations 1 and 2 showed a comparable macrophage phenotype. In direction 1, population 3 exhibited a notable shift to a pro‐inflammatory phenotype. In direction 2, there was a slight shift in population 4 and a remarkable shift in population 5 toward a pro‐inflammatory phenotype (Figure 4d). To further understand the function of each KC population, we analyzed KC population marker genes. It is noteworthy that most ARKC genes identified in Figure 3j,k, such as *Cd83*, *Dapk1*, *Got1*, *Hbegf*, *Rhob*, and *Trim25*, were found in the population 5 marker gene set. Populations 4 and 5 showed a comparable expression pattern of their marker genes (Figure 4e). Pathway analysis revealed that the population 1 marker gene set was involved in adherens junction and focal adhesion signaling pathways; the population 2 marker gene set was enriched in thermogenesis, protein precession, and ribosome signaling pathways. The marker genes in population 3 mainly regulated antigen processing and presentation, phagosome, chemokine, and NF‐kappa B signaling pathways. The populations 4 and 5 cell marker gene set was significantly clustered into pro‐inflammatory pathways, including TNF, IL‐17, NF‐kappa B, and chemokine signaling pathways (Figure 4f). As compared to populations 1 and 2, the ARKC genes were significantly upregulated in KC populations 4 and 5, including *Got1*, *Dapk1*, *Trim25*, *Hbegf*, *Dyrk2*, *Abca1*, *Lpcat2*, *Cd83*, and *Bcl2a1b*. However, the expression levels of *Got1*, *Dapk1*, *Trim25*, *Hbegf*, *Dyrk2*, and *Abca1* were significantly decreased in KC population 3, as compared to KC populations 1 and 2 or 4 and 5 (Figure 4g; Figure S4A–G). In KC populations 4 and 5, the expression of ARKC genes, such as *Got1*, *Dapk1*, *Dyrk2*, *Rhob*, *Hbegf*, *Cd83*, *Lpcat2*, *Ncoa4*, *Hes1*, and *Bcl2a1b*, showed a highly positive correlation with the expression of chemokine and pro‐inflammatory cytokine genes, including *Ccl2*, *Tnf*, and *Il1b*. However, the expression of *Vcam1* was negatively correlated with the expression of chemokine and pro‐inflammatory cytokine genes (Figure 4h). In contrast to KC populations 4 and 5, the expression levels of ARKC genes, including *Rhob*, *Egr1*, *Lpcat2*, *Dyrk2*, *Hbegf*, and *Ncoa4*, were negatively correlated with pro‐inflammatory cytokine gene expression in KC population 3 (Figure S4H). Instead, in KC population 3, the expression of genes responsible for antigen processing and presentation showed a strong positive correlation with chemokine and pro‐inflammatory cytokine gene expression (Figure 4i).

In MDMs, the terminus of cell trajectory is majorly composed of the old MDMs (Figure 4j,k). We divided MDMs into three populations based on their pseudotime, including population 1 (early stage), population 2 (middle stage), and population 3 (late stage) (Figure 4l). MPI analysis showed that MDM population 3 had a less pro‐inflammatory phenotype compared to MDM population 1 or 2 (Figure S4I). We further analyzed MDM population marker genes and found that MDM population 1 marker genes were involved in focal adhesion and adherens junction. MDM population 2 marker genes were enriched in phagosome, antigen processing and presentation, chemokine signaling pathway, and phagocytosis, whereas MDM population 3 marker genes were related to cycle, RNA degradation, and nucleotide metabolism; this indicates that MDM population 3 is quiescent in antigen processing and presentation as well as pathogen clearance (Figure 4m,n). Collectively, these results indicate that aging promotes pro‐inflammation in KCs and induces functional quiescence in MDMs.

### 2.5 FOXO1 inhibition improves glucose homeostasis and attenuates inflammation in old mice

**FIGURE 5:**
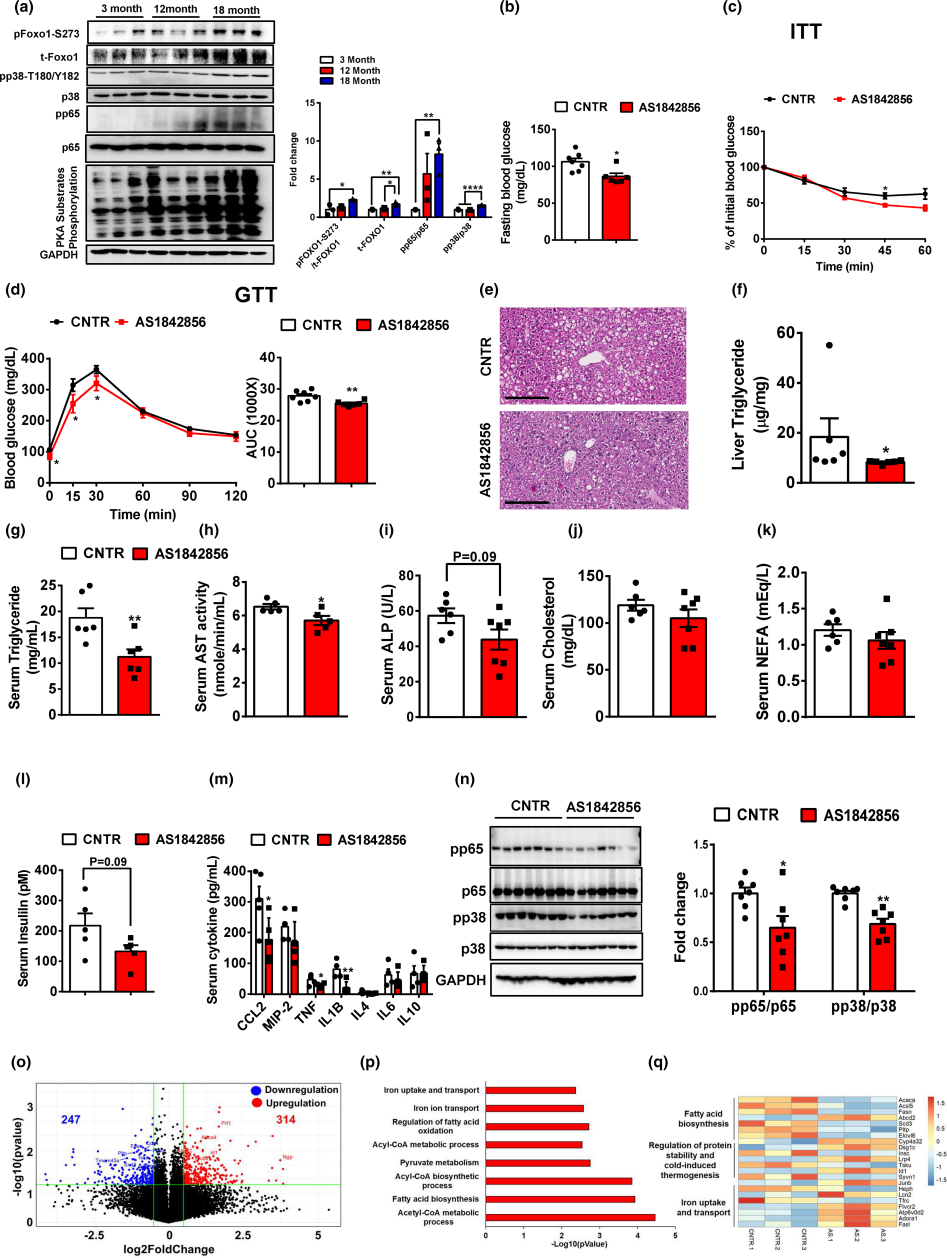
FOXO1 inhibition improves glucose homeostasis, hepatic steatosis, and chronic inflammation in old mice. (a) FOXO1 activity was detected in the livers of young and old mice, *n* = 3 mice/group. (b) Overnight (16 h) fasting blood glucose in the old mice treated with AS1842856, *n* = 7 mice/group. (c) Insulin tolerance tests in control and AS1842856‐treated old mice, *n* = 7 mice/group. (d) Glucose tolerance tests in control and AS1842856‐treated old mice, *n* = 7 mice/group. (e) H&E staining of the livers of control and AS1842856‐treated old mice. Representative images are shown. (f) Liver triglycerides in control and AS1842856‐treated old mice, *n* = 6‐7 mice/group. (g–l) Serum triglycerides, AST, ALP, cholesterol, NEFA, and insulin in control and AS1842856‐treated old mice, *n* = 5‐7 mice/group. (m) Serum cytokines in control and AS1842856‐treated old mice, *n* = 4–5 mice/group. (n) The phosphorylation of p38 and p65 in the livers of control and AS1842856‐treated old mice, *n* = 7 mice/group. (o) Volcano plot of hepatic genes in control and AS1842856‐treated old mice analyzed by RNA‐Seq. (p) Pathway analysis of differentially expressed genes between control and AS1842856‐treated mouse livers. (q) Heatmap of representative differentially expressed genes between control and AS1842856‐treated mouse livers. All data are presented as mean ± SEM. * *p*<0.05, ** *p*<0.01, **** *p*<0.0001.

Given that the FoxO signaling pathway is significantly enhanced in the old liver macrophages, we further detected FOXO1 activity in the livers of old mice. We found that FOXO1 activity was significantly enhanced in the mouse liver during aging, as indicated by significant increases in phosphorylated FOXO1 at S273 (pFOXO1‐S273) by 130%, total FOXO1 (t‐FOXO1) by 75%, and PKA substrate phosphorylation. Liver inflammation was also aggravated during aging, as indicated by significant increases in phosphorylated p38 (pp38) by 75% and phosphorylated p65 (pp65) by 60% (Figure 5a). These results indicate that FOXO1 potentially plays an important role in the regulation of the liver aging process. To detect the role of FOXO1 in liver aging, we treated the old mice (18‐month‐old) with FOXO1 inhibitor, AS1842856 (10 mg/kg body weight), through daily oral gavage for 5 weeks. FOXO1 inhibition had no significant effect on body weight, liver weight, epididymal white adipose tissue (eWAT) weight, and inguinal white adipose tissue (iWAT) weight in old mice (Figure S5A). The fat mass and lean mass in old mice were not affected by FOXO1 inhibitor treatment (Figure S5B). The old mice treated with FOXO1 inhibitor had an 18% decrease in overnight fasting blood glucose (Figure 5b). FOXO1 inhibition significantly improved glucose tolerance and insulin sensitivity in old mice (Figure 5c,d). Liver fat accumulation was significantly attenuated in old mice treated with FOXO1 inhibitor (Figure 5e; Figure S5C). Consistently, liver and serum triglycerides were significantly decreased (by 40% and 54%, respectively) in the old mice treated with FOXO1 inhibitor (Figure 5f,g). In old mice, FOXO1 inhibition led to a significant decrease in serum AST and nonsignificant decreases in serum ALP, cholesterol, NEFA, HDL, and insulin (Figure 5h–l; Figure S5D). Serum ALT and LDL were not changed by FOXO1 inhibition in old mice (Figure S5E,F).

We further analyzed serum cytokine levels and found that serum CCL2, TNF, and IL1B levels were significantly decreased (by 43%, 41%, and 75%, respectively) in old mice treated with FOXO1 inhibitor, compared to the control old mice (Figure 4m). Consistently, FOXO1 inhibition significantly attenuated inflammation in the old mouse livers, as indicated by decreases in pp65 and pp38 by 35% and 31%, respectively (Figure 5n). We further detected the pro‐inflammatory response in the peritoneal macrophages (PMs) of old mice. Consistently, FOXO1 inhibitor treatment significantly reduced gene expression levels of *Tnf* by 39%, *Il1β* by 33%, and *Il6* by 56% in the PMs of old mice (Figure S5G). We then analyzed gene expression profiles in the livers of control and FOXO1 inhibitor treated old mice using RNA‐Seq. We identified around 561 differentially expressed genes (Upregulation: 314 and Downregulation: 247; Figure 5o). Pathway analysis showed that the differentially expressed gene set was involved in fatty acid biosynthesis, iron ion transport, and fatty acid oxidation signaling pathways (Figure 5p). Of note, fatty acid biosynthesis was significantly attenuated by FOXO1 inhibition in the liver of old mice, which potentially contributes to the improvement in liver fat deposition (Figure 5q). These results indicate that FOXO1 inhibition improves glucose homeostasis, decreases liver fat accumulation, and attenuates inflammation in old mice.

### 2.6 FOXO1 inhibition improves pro‐inflammation in hepatic macrophages during aging

**FIGURE 6:**
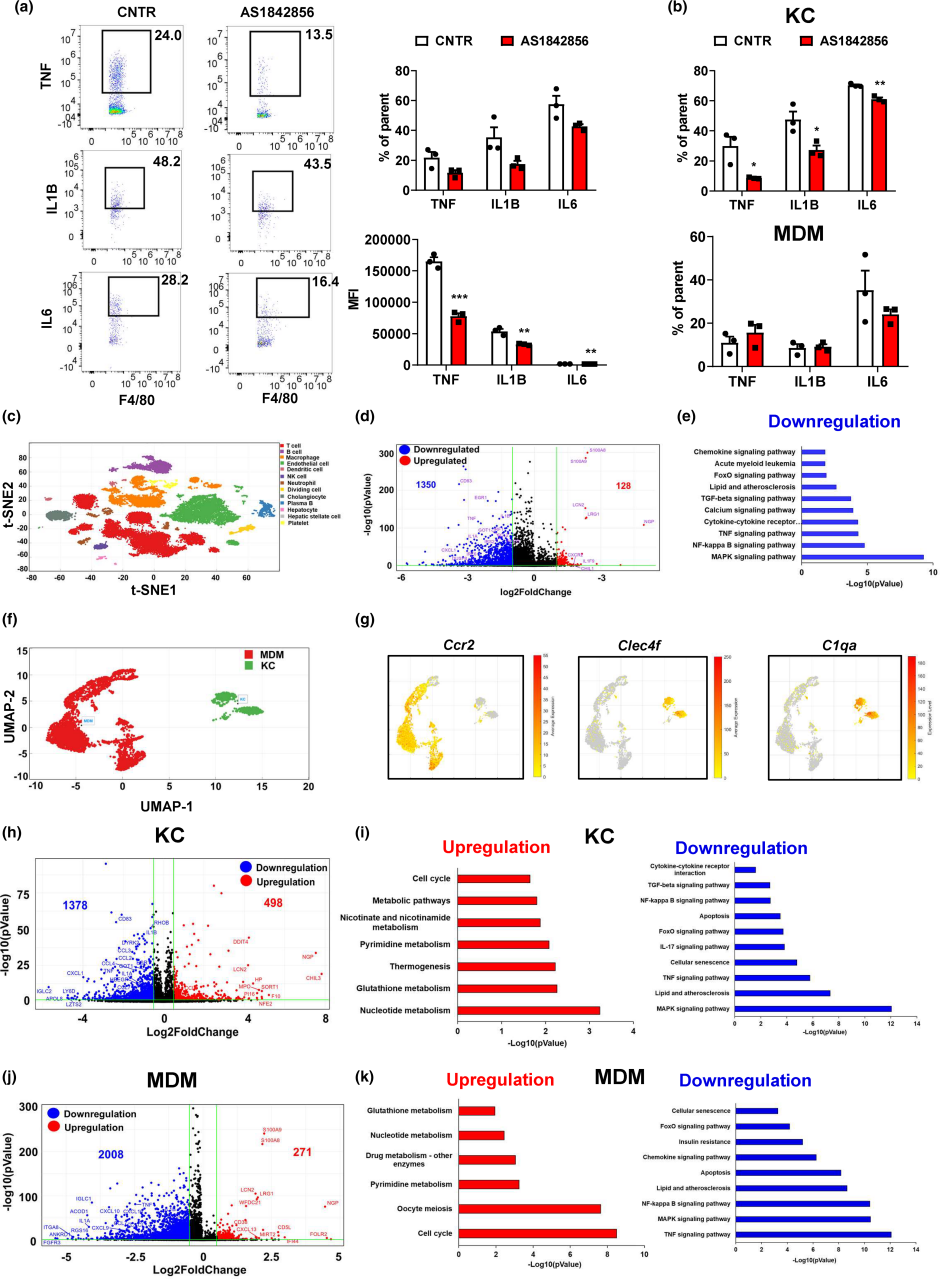
FOXO1 inhibition improves pro‐inflammation in hepatic macrophages during aging. (a) The percentage and MFI of intracellular pro‐inflammatory cytokines in the hepatic macrophages of control and AS1842856‐treated old mice, *n* = 3 mice/group. (b) The percentage of intracellular pro‐inflammatory cytokines in the KCs and MDMs of control and AS1842856‐treated old mice, *n* = 3 mice/group. (c) t‐SNE visualization of liver cell clusters from control and AS1842856‐treated old mice. (d) Volcano plot of gene expression in the hepatic macrophages of control and AS1842856‐treated old mice. (e) Pathway analysis of genes significantly downregulated by AS1842856 treatment in old hepatic macrophages. (f) Illustration of liver‐resident KCs (green) and MDMs (red) in control and AS1842856‐treated old mice. (g) Feature plot of KC and MDM marker gene expression in control and AS1842856‐treated old mice. (h) Volcano plot of gene expression in the KCs of control and AS1842856‐treated old mice. (i) Pathway analysis of differentially expressed genes between control and AS1842856‐treated old mouse KCs. Red: upregulated genes in the KCs of AS1842856‐treated old mice; Blue: downregulated genes in the KCs of AS1842856‐treated old mice. (j) Volcano plot of gene expression in the MDMs of control and AS1842856‐treated old mice. (k) Pathway analysis of differentially expressed genes between control and AS1842856‐treated old mouse MDMs. Red: upregulated genes in the MDMs of AS1842856‐treated old mice; Blue: downregulated genes in the MDMs of AS1842856‐treated old mice. All data are presented as mean ± SEM. *p < 0.05, **p < 0.01. ***p < 0.001.

Considering that aging induces pro‐inflammatory response in hepatic macrophages, we then detected the effect of FOXO1 inhibition on pro‐inflammation in the hepatic macrophages of old mice using flow cytometry. As compared to control old mice, AS1842856‐treated old mice showed a decreasing trend in the intensity of intracellular pro‐inflammatory cytokines and significant reduction in the MFI of intracellular pro‐inflammatory cytokines in hepatic macrophages (Figure 6a). Consistently, in bone marrow‐derived macrophages (BMDMs), FOXO1 inhibitor treatment significantly attenuated LPS‐induced mRNA expression levels of *Tnf* by 90% and *Il1b* by 88% (Figure S6A). Considering that aging differentially affects the phenotypes of KCs and MDMs, we further detected pro‐inflammatory cytokine levels in KCs and MDMs. In old KCs but not MDMs, FOXO1 inhibition significantly decreased percentage and MFI of intracellular signal of TNF, IL1B, and IL6 (Figure 6b; Figure S6B). We further performed liver NPC scRNA‐Seq to analyze the effect of FOXO1 inhibition on hepatic macrophage features during aging at the single‐cell level. We obtained a total of 27,839 single‐cell transcriptomes from the control and AS1842856‐treated old mouse livers. T‐SNE visualization of the combined data revealed 13 clusters, which correspond to T cells, B cells, macrophages, endothelial cells, dendritic cells, NK cells, neutrophils, dividing cells, cholangiocytes, plasma B, hepatocytes, HSC, and platelets, based on their marker gene expression (Figure 6c; Figure S6C). We further analyzed macrophage cluster and found that most of genes (~1350) were downregulated and only 128 genes were upregulated by FOXO1 inhibition in the hepatic macrophages of old mice (Figure 6d). The downregulated gene set was mainly involved in pro‐inflammatory pathways, including MAPK, NF‐kappa B, TNF, and chemokine signaling pathways. As expected, FOXO1 inhibition decreases the activity of the FoxO signaling pathway (Figure 6e). The upregulated gene set was enriched in cell cycle and meiosis functions (Figure S6D). The expression levels of pro‐inflammatory cytokines, including *Ccl2*, *Tnf*, *Il1b*, and *Il1a*, were significantly decreased in the hepatic macrophages of old mice treated with AS1842856 (Figure S6E). We then divided hepatic macrophages into KC and MDM populations based on their marker gene expression (Figure 6f,g). In old KCs, FOXO1 inhibition led to 498 upregulated and 1378 downregulated genes (Figure 6h). The upregulated gene set was involved in metabolic pathways, including nucleotide metabolism, glutathione metabolism, and thermogenesis. Consistently, FOXO1 inhibition significantly attenuated the activity of pro‐inflammatory pathways, such as MAPK, NF‐kappa B, TNF, and chemokine signaling pathways, as well as the FoxO signaling pathway in old KCs (Figure 6i). The expression levels of pro‐inflammatory cytokine genes, such as *Tnf*, *Il1b*, and *Ccl2*, and ARKC genes including *Got1*, *Dyrk2*, *Dapk1*, *Rhob*, and *Cd83* were significantly downregulated by FOXO1 inhibition in old KCs (Figure S6F). In old MDMs, FOXO1 inhibition led to 271 upregulated genes and 2008 downregulated genes (Figure 6j). The gene upregulated by FOXO1 inhibition in old MDMs were clustered into cell cycle and nucleotide metabolism pathways. In line with results in KCs, FOXO1 inhibition attenuated the activity of pro‐inflammatory pathways in old MDMs, including TNF, MAPK, NF‐kappa B, and chemokine signaling pathways. As expected, FOXO1 inhibition blocked the FoxO signaling pathway (Figure 6k). Similarly, FOXO1 inhibition significantly decreased expression levels of pro‐inflammatory cytokine genes, such as *Ccl2*, *Tnf*, *Il1b*, and *Il1a*, and ARKC genes including *Got1*, *Dyrk2*, *Dapk1*, and *Cd83*, in old MDMs (Figure S6G). These results indicate that FOXO1 inhibition decreases aging‐induced pro‐inflammation in hepatic macrophages.

### 2.7 FOXO1 inhibition rescues aging‐induced phenotypes in KCs but not in MDMs

**FIGURE 7:**
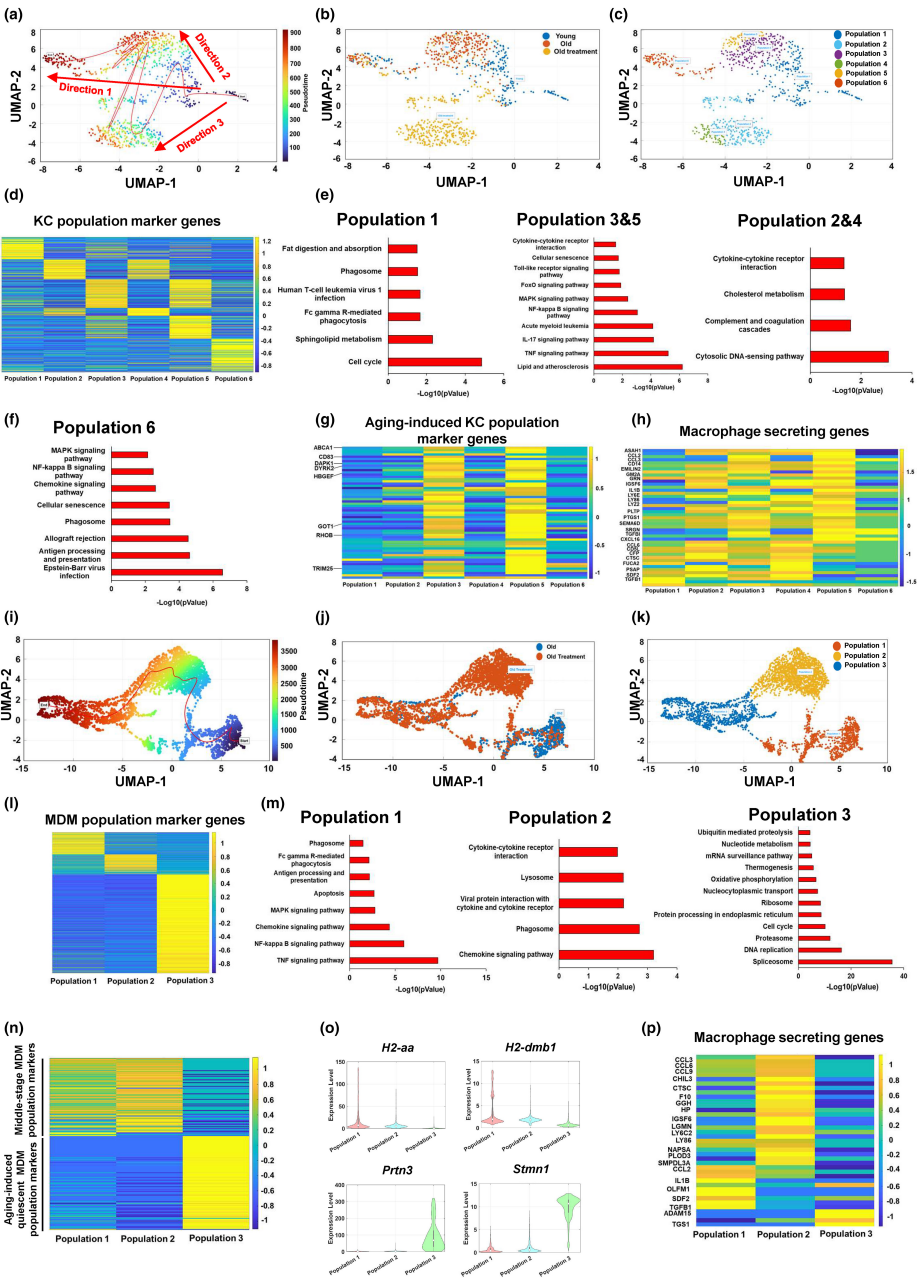
FOXO1 inhibition rescues aging‐induced phenotypes in KC but not in MDM. (a) Pseudotime analysis of the KCs from young, control old, and AS1842856‐treated old mice. (b) Illustration of the KCs from young, control old, and AS1842856‐treated old mice. (c) The KCs were divided into 6 populations based on their pseudotime analysis. (d) Heatmap of KC population marker genes. (e, f) Pathway analysis of KC population marker genes. (g) Heatmap of aging‐responsive genes in KCs. (h) Heatmap of hepatic macrophage‐secreted factors in KCs. (i) Pseudotime analysis of the MDMs from control and AS1842856‐treated old mice. (j) Illustration of the MDMs from control and AS1842856‐treated old mice. (k) The MDMs were divided into three populations based on their pseudotime analysis. (l) Heatmap of MDM population marker genes. (m) Pathway analysis of MDM population marker genes. (n) Heatmap of young and old MDM marker genes. (o) Feature plots of young and old MDM marker genes. (p) Heatmap of macrophage‐secreted factors in MDMs.

To further analyze the effect of FOXO1 inhibition on aging‐induced phenotypes in KCs and MDMs, we performed pseudotime analysis. In KCs, there appeared to be three different directions (Figure 7a). The terminus of KC direction 1 consisted of KCs from young, control old, and AS1842856‐treated old mouse livers. However, the terminus of KC direction 2 only contained KCs from control old mouse livers, and the terminus of KC direction 3 only presented KCs from AS1842856‐treated old mouse livers (Figure 7b). We then separated KCs into six different populations based on their pseudotime (Figure 7c and Table 1). To further understand the functions of the different populations, we analyzed the population marker genes and found that population 2 exhibited a similar population marker pattern to population 4, with both being located at direction 3. Populations 3 and 5 were located at direction 2 and showed a comparable population marker pattern (Figure 7d). Pathway analysis of population marker genes revealed that the marker genes in population 1, consisting of early‐stage KCs, were mainly involved in cell cycle, phagocytosis, and phagosome pathways. In populations 3 and 5 (direction 2; indicated as the aging‐responsive KC populations), their marker genes were significantly enriched in pro‐inflammatory pathways, including TNF, NF‐kappa B, and MAPK signaling pathways. Populations 2 and 4 (Direction 3) only contained KCs from AS1842856‐treated old mouse liver and their marker genes were less pro‐inflammatory than that in populations 3 and 5, as indicated by their enrichments in cytosolic DNA‐sensing and complement and coagulation pathways (Figure 7e). We then analyzed the expression of the aging‐induced KC population marker genes identified in Figure 4e. KC populations 3 and 5 showed high expression levels of these marker genes. However, KC populations 2 and 4 showed a significant downregulation of these marker genes and exhibited a similar pattern to population 1 that is indicated as an early stage (Figure 7g). Further analysis of KC secretum profiles revealed that AS1842856‐treated populations 2 and 4 exhibited a comparable secretum profile to early‐stage KC population 1, and showed a different secretum profile to KC populations 3 and 5 (Figure 7h).

**TABLE 1:** Summary of three different directions in KCs based on their pseudotime.

Directions	Populations	Stages	Contained groups	Marker gene pathways
1	1	Early stage of all directions	Young, control old, and AS1842856‐treated old KCs	Cell cycle, phagocytosis, and phagosome pathways
	6	Late stage		Pro‐inflammatory pathways, phagosome, antigen processing and presentation
2	3	Mid‐stage	Control old KCs	Pro‐inflammatory pathways
	5	Late stage		
3	2	Mid‐stage	AS1842856‐treated old KCs	Cytosolic DNA‐sensing and complement and coagulation pathways
	4	Late stage		

In MDMs combined from the livers of control and AS1842856‐treated old mice, pseudotime analysis showed that there appeared to be only one direction (Figure 7i). The terminus of MDM trajectory is composed of MDMs from both control and AS1842856‐treated old moues livers. The majority of AS1842856‐treated old MDMs showed a shift from control old MDMs (Figure 7j). According to their pseudotime, we divided MDMs into three populations, including population 1 (early stage), population 2 (middle stage), and population 3 (late stage; Figure 7k and Table 2). We further performed population marker gene analysis and found that MDM population 1 was characterized by pro‐inflammatory pathways, antigen processing and presentation, and phagosome function. Compared to MDM population 1, MDM population 2 showed less pro‐inflammatory but still maintained phagosome function. MDM population 3 showed similar features to the functionally quiescent population induced by aging (Figure 7l,m). To further detect the effect of FOXO1 inhibitor on aging‐induced MDM features, we analyzed the expression pattern of the middle‐stage MDM and aging‐induced quiescent MDM population marker genes identified in Figure 4m. We found that MDM populations 1 (control MDM) and 2 (AS1842856‐treated MDM) exhibited a similar pattern, with a high expression of middle‐stage MDM population marker genes and a low expression of aging‐induced quiescent MDM population marker genes; however, MDM population 3 (both control and AS1842856‐treated MDM) showed a high expression in aging‐induced quiescent MDM population marker genes, suggesting that FOXO1 inhibition has no significant effect on aging‐regulated gene expression in MDM (Figure 7n). The expression levels of genes responsible for antigen process and presentation were significantly decreased in MDM population 3 (Figure 7o; Figure S7A). The marker genes of MDM population 3, including *Stmn1*, *Prtn3*, *Birc5*, *Pclaf*, and *Mcm6*, were significantly upregulated in MDM population 3 (Figure 7o; Figure S7B). In addition, the MDM populations 1, 2, and 3 showed different secretum profiles, especially in populations 1 and 2. The expression levels of pro‐inflammatory cytokine genes, including *Ccl2* and *Il1b*, were significantly decreased by FOXO1 inhibition in MDM population 2, compared to MDM population 1 (Figure 7p). Taken together, these results indicate that FOXO1 inhibition improves aging‐induced pro‐inflammation in KCs but has a limited effect on aging‐induced functional quiescence in MDMs.

**TABLE 2:** Summary of three populations in MDMs based on their pseudotime.

Populations	Stages	Contained groups	Marker gene pathways
1	Early stage	Control old, and AS1842856‐treated old MDMs	Pro‐inflammatory pathways, antigen processing and presentation, and phagosome function
2	Mid‐stage	AS1842856‐treated old MDMs	Phagosome function, cytokine, chemokine signaling pathway
3	Late stage	Control old, and AS1842856‐treated old MDMs	Cell cycle, oxidative phosphorylation, DNA replication pathways

## 3 DISCUSSION

Age is an important risk factor for numerous chronic diseases, such as cardiovascular diseases, diabetes, and cancer. Inflamm‐aging is a condition characterized by increased levels of blood inflammatory markers in aging individuals, contributing to the development of chronic diseases. In this study, we investigated the characteristics of the aging liver and examined the effects of FOXO1 on aging‐induced liver inflammation and glucose dysregulation. We found that aging promoted glucose intolerance, liver fat deposition, and systemic inflammation in mice. Liver macrophages, including KCs and MDMs, were differentially regulated during aging. In KCs, aging enhanced pro‐inflammation, while aging induced a shift to a functionally quiescent stage in MDMs. The activity of FOXO1 was stimulated in the old mouse livers. FOXO1 inhibition by AS1842856 significantly improved glucose homeostasis, liver fat accumulation, and systemic inflammation in old mice. Furthermore, FOXO1 inhibition decreased aging‐induced pro‐inflammation in KCs but had a limited effect on aging‐induced functional quiescence in MDMs. These results indicate that FOXO1 is a key player in mediating aging‐induced inflammation in liver macrophages, especially KCs, and glucose homeostasis dysregulation.

The metabolic syndrome of aging is also called insulin resistance syndrome, indicating that insulin resistance is a key feature in the aging‐induced metabolic syndrome (Barzilai & Ferrucci, 2012). In our study, we found that insulin sensitivity and glucose homeostasis were significantly impaired in old mice. The systemic inflammation was significantly enhanced in old mice, as indicated by a significant increase in chemokine CCL2 and pro‐inflammatory cytokines TNF and IL1B, and a decrease in anti‐inflammatory cytokine IL10; this enhancement in pro‐inflammation contributes to the aging‐induced insulin resistance. Moreover, we observed a significant increase in liver lipid accumulation in old mice, further contributing to the development of insulin resistance. Consistently, RNA‐Seq results revealed that lipid biosynthesis was enhanced, and fatty acid oxidation was attenuated in the liver of old mice. In addition, we found that peroxisome and ferroptosis pathways were activated during aging. The oxidative organelle peroxisome promotes ferroptosis susceptibility and evasion (Zou et al., 2020). Thus, liver peroxisome is stimulated to promote ferroptosis during aging, thereby leading to oxidative stress, affecting liver function, and contributing to the process of aging. The excessive cellular iron accumulation is one the mechanisms of ferroptosis (Cao & Dixon, 2016). Although iron deficiency anemia is prevalent in old age (Fairweather‐Tait et al., 2014), we found that expression of *transferrin receptor* (*Tfrc*) was significantly upregulated in the old mouse livers; these results indicate that liver iron level is potentially increased during aging, thereby contributing to the liver ferroptosis. However, the effect of aging on iron homeostasis in liver, especially in different liver cells, needs to be further investigated. Cellular senescence is characterized by an irreversible cell‐cycle arrest, which is triggered by stress‐related factors and physiological processes (Gorgoulis et al., 2019). Cellular senescence promotes aging‐induced hepatic steatosis by impairing the ability of mitochondria to metabolize fatty acids (Ogrodnik et al., 2017). In this study, we found that cellular senescence was stimulated in the liver of old mice, which potentially contributes to the aging‐induced hepatic steatosis. Senescent cells secrete a plethora of inflammatory cytokines, chemokines, and proteases; these are collectively termed the senescence‐associated secretory phenotype (SASP) (Gorgoulis et al., 2019). Therefore, aging‐induced liver senescence also contributes to systemic and liver inflammation.

The majority of older individuals develop inflamm‐aging characterized by elevated levels of pro‐inflammatory markers in tissues (Ferrucci & Fabbri, 2018). Macrophages play important roles in mediating inflammation in metabolic diseases (Chawla et al., 2011). Considering that metabolic inflammation shares similar features with inflamm‐aging in terms of clinical presentation and pathogenic factors (Prattichizzo et al., 2018), macrophages play a potentially key role in the regulation of inflamm‐aging. In our study, we analyzed the effect of aging on the features of liver macrophages at the single‐cell level. Pro‐inflammation was significantly enhanced in the hepatic macrophages of old mice, as indicated by significant increases in the expression of pro‐inflammatory cytokine genes *Tnf*, *Il1b*, and *Ccl2*. It is noteworthy that in liver, macrophages are the major source of pro‐inflammatory cytokines and chemokines including TNF, IL1B, and CCL2; this result suggests that liver macrophages play a key role in liver inflamm‐aging. Cell–cell crosstalk network analysis showed that macrophages interacted with hepatocytes through the TNF, CCL2/3, and IL1B signaling pathways. The activation of the TNF and IL1B signaling pathways promotes insulin resistance and fat deposition in hepatocytes (Chung et al., 2015; De Taeye et al., 2007; Nov et al., 2010; Wandrer et al., 2020). In liver, CCL2 plays an important role in regulating liver pathology and modulating liver disease progression (She et al., 2022). Thus, the increased secretion of TNF, IL1B, and CCL2 in the hepatic macrophages of old mice potentially aggravates insulin resistance, liver steatosis, and the progression of chronic liver diseases. The immune cells in liver protect against pathogen invasions. The immune cells are significantly changed during aging; for example, aged neutrophils exhibiting impaired respiratory burst and reactive nitrogen intermediates, aged dendritic cells showing failure to stimulate T and B cells, and NKs from the elderly showing less ability to destroy tumor cells (Plackett et al., 2004). We found that the CCL2/3, CXCL9/10, and HBEGF signaling pathways mainly mediated the crosstalk between macrophages and other liver immune cells, including NK cells, dividing cells, T cells, B cells, dendritic cells, neutrophils, and plasma B. It is reasonable to speculate that aging‐upregulated expression of *Ccl2*, *Ccl3*, *Cxcl10*, and *Hbegf* in hepatic macrophages may contribute to aging‐induced changes in liver immune cells.

Hepatic macrophages are a heterogenous population composed of KCs and MDMs (Stahl et al., 2018). KCs are primitive cells primarily derived from the yolk sac (Gomez Perdiguero et al., 2015) and MDMs are derived from circulating monocytic progenitor (Holt et al., 2008); this suggests that KCs and MDMs may have different functions in liver. A previous study reported that KCs and MDMs have distinct morphologies and transcriptional profiles in fatty livers (McGettigan et al., 2019). Consistently, our study showed that KCs and MDMs had different secretum profiles and their functions were differentially regulated during aging. The number of KCs, but not MDMs, was significantly increased in liver during aging; this is in line with previous reporting (Hilmer et al., 2007). The mechanisms of KC renewal are not clearly understood. There are two hypotheses about KC renewal: 1) KCs come from bone marrow‐derived monocytes; 2) KCs originate from local intrahepatic progenitors and proliferate as mature cells (Diesselhoff‐Den Dulk et al., 1979; Jenkins et al., 2011; Klein et al., 2007; Nguyen‐Lefebvre & Horuzsko, 2015). We found that the cell cycle pathway was attenuated, and apoptosis was enhanced in KCs during aging. These results indicate that aging increases the number of KCs, potentially by increasing the differentiation of monocytes or intrahepatic progenitors into KCs. In addition, we found that aging significantly increased pro‐inflammation in KCs but not MDMs. Thus, aging induces pro‐inflammation of hepatic macrophages mainly through targeting KCs. In our study, we identified aging‐responsive KC‐specific (ARKC) genes, such as *Cd83*, *Dapk1*, *Dyrk2*, *Got1*, *Hbegf*, *Rhob*, *Trim25*, and *Lpcat2*. Pseudotime analysis showed that KCs were also a heterogenous population and there were two directions in KCs (Directions 1 and 2). We found that Direction 1 KCs were responsible for scavenger function, whereas Direction 2 KCs, an aging‐induced population, showed a significant increase in pro‐inflammation during aging. ARKC gene expression levels were positively correlated with pro‐inflammation cytokine genes in the KCs of old mouse livers; this suggests an important function of ARKC genes in aging‐induced pro‐inflammation in KCs. However, the roles of ARKC genes in inflamm‐aging need to be further investigated.

KCs are located along the hepatic sinusoidal endothelial cells and function as a scavenger to clear pathogens from the blood (Tacke & Zimmermann, 2014). During liver injury, KCs are activated to release chemokines, thus recruiting circulating monocytes into inflamed areas. The infiltrated monocytes differentiate into macrophages involved in liver injury development and restoration (Krenkel & Tacke, 2017). Although pro‐inflammatory cytokines or chemokines are significantly increased in the KCs of old mouse livers, we did not observe an increase in the infiltration of monocytes, indicated by the number of MDMs. We found that mRNA expression of the *Ccr2* gene responsible for monocyte recruitment was significantly decreased in the old MDMs; this may explain why MDM number is not altered during aging. Pseudotime analysis indicated an aging‐induced MDM population that showed a functionally quiescent phenotype with a decreased phagocytosis capacity. The infiltrated MDMs are involved in the phagocytosis of dead cells and the restoration of the extracellular matrix (Krenkel & Tacke, 2017; Zigmond et al., 2014). The impaired phagocytosis ability in old MDMs potentially contributes to aging‐enhanced vulnerability to acute liver injury.

FOXO1, a forkhead transcription factor, plays an important role in the regulation of energy and nutrient homeostasis (Cheng et al., 2009; Kousteni, 2012; Wu et al., 2018; Zhang et al., 2012, 2019). In our study, we found that the FoxO signaling pathway was significantly enhanced in the hepatic macrophages of old mice. Consistently, FOXO1 protein levels were significantly increased in the liver of old mice. However, we did not observe significant changes in *Foxo1* mRNA levels. These results indicate that the upregulation of FOXO1 protein is potentially attributed to the post‐translational modification. In our previous studies, we showed that PKA or p38α phosphorylated FOXO1 at S273, thus increasing FOXO1 protein stability and promoting its nuclear translocation (Wu et al., 2018; Yang, Liao, et al., 2023). We did observe that PKA activity, pp38, and pFoxo1‐S273 were significantly increased in the livers of old mice. In addition, the increased activity of p65 in old liver enhances cAMP‐PKA signaling pathway through suppression of PDE3B (Ke et al., 2015). Therefore, aging‐stimulated FOXO1 activity promotes gluconeogenesis, increases fasting blood glucose, and impairs glucose homeostasis. The AS1842856 compound binds to FOXO1 and reduces its activity without affecting its transcription and protein expression (Nagashima et al., 2010). In our study, we found that AS1842856 treatment significantly improved glucose homeostasis and hepatic steatosis in old mice. Our previous report showed that inactivation of FOXO1 improved hepatic mitochondrial function (Cheng et al., 2009; Yang et al., 2019), which may contribute to the lipid clearance in liver. In addition, we observed the upregulation of fatty acids oxidation gene *Cpt1α* and downregulation of lipogenic gene *Fasn* in the livers of old mice treated with AS1842856. However, whether FOXO1 regulates *Cpt1α* and *Fasn* mRNA expression directly or indirectly remains unknown. Iron transport gene *Tfrc* was significantly downregulated in the livers of old mice; this result indicates that FOXO1 inhibition might decrease hepatic iron levels and attenuate iron‐dependent ferroptosis in liver.

In this study, we found that liver and systemic inflammatory levels were significantly improved in old mice treated with AS1842856. In both PMs and hepatic macrophages of old mice, FOXO1 inhibition decreased pro‐inflammation; this result is in line with the previous report that myeloid FOXO1 deficiency favors macrophage M2 polarization (Lee et al., 2022). We found that FOXO1 deficiency significantly reduced pro‐inflammation in both KCs and MDMs, suggesting that AS1842856 targets the hepatic macrophages without selectivity. Pseudotime analysis showed that FOXO1 inhibition reversed the aging‐induced pro‐inflammation in KCs, whereas aging‐induced MDM functional quiescence was not affected by FOXO1 inhibition. Thus, FOXO1 inhibition exerts an anti‐aging effect in KCs. FOXO1 promotes inflammation in mature macrophages through the activation of TLR4‐ and STAT6‐mediated signaling pathways (Fan et al., 2010; Lee et al., 2022). However, the roles of TLR4‐ and STAT6‐mediated signaling pathways in aging‐induced pro‐inflammation in KCs require further study. Of note, FOXO1 inhibition significantly decreased the expression levels of ARKC genes in both KCs and MDMs of old mice. Therefore, the activation of FOXO1 plays an important role in the aging‐induced pro‐inflammation in hepatic macrophages. However, the underlying mechanism of how FOXO1 regulates the expression of ARKC genes in hepatic macrophages also needs further investigated. Collectively, our results indicate that FOXO1 is a key player in mediating aging‐induced inflammation and nutrient homeostasis dysregulation; FOXO1 could be a potential therapeutic target in preventing inflamm‐aging and aging‐induced chronic diseases.

## 4 EXPERIMENTAL PROCEDURES

### 4.1 Animals

All animal protocols were approved by the Institutional Animal Care and Use Committee of Texas A&M University. All mice were housed at 22°C in a 12 h light and 12 h dark cycle with ad libitum access to food and water. Male mice were used in all the experiments. The old mice (18‐month‐old) were administered with corn oil or AS1842856 (10 mg/kg body weight) (Cayman Chemical) through daily oral gavage for 5 weeks. The body composition of mice was measured using EchoMRI. The mice were terminated at random fed condition after 5 weeks treatment of AS1842856.

### 4.2 Insulin/glucose tolerance tests

For insulin tolerance tests, mice were fasted for 5 h and administered with 1 U/kg insulin through intraperitoneal (i.p.) injection. Blood glucose was monitored. For glucose tolerance tests, 15% glucose was i.p. injected into mice after 16 h fasting. Blood glucose was monitored at indicated time points.

### 4.3 Isolation and scRNA‐Seq analysis of liver NPCs

Liver NPCs were isolated following a two‐step protocol of pronase/collagenase digestion (Xiong et al., 2019). Briefly, liver was perfused with a calcium‐free Hank's Balanced Salt Solution (HBSS) supplemented with 0.075% NaHCO3 and 0.2 mg/mL EDTA. Then the liver was digested by sequential perfusion with 0.4 mg/mL pronase (Sigma) and 0.2% collagenase type II (ThermoFisher). The liver was minced and then incubated in HBSS with 0.2% collagenase type II, 0.4 mg/mL pronase, and 0.1 mg/mL DNase (Roche) at 37°C for 10 min. After terminating the digestion with DMEM with 10% FBS, the liver cell suspension was centrifuged at 50 *g* for 3 min to discard hepatocytes, and then centrifuged at 500 *g* for 7 min to collect NPCs. After treatment with RBC lysis, the NPC suspension was centrifuged, resuspended in HBSS, and subjected to density gradient centrifugation with 10% and 40% Opti‐prep. The resulting NPCs were used to perform scRNA‐Seq analysis using 10X Genomics Chromium Single‐Cell 3′. The scRNA‐Seq was performed at the TIGSS molecular genomics core in Texas A&M University.

### 4.4 Liver NPC single‐cell RNA sequencing

The Cell Ranger Software Suite (v6.1.2) was used to perform sample de‐multiplexing, process the barcode, and count the single‐cell 5′ unique molecular identifier (UMI). For each sample, a gene‐by‐cell matrix was generated with default parameters. To filter out low‐quality cells, cells with >15% of UMIs assigned to mitochondrial genes were excluded. Genes expressed in <2% of cells were excluded. To normalize the data, the number of UMIs in each cell was divided by the total counts in each cell and multiplied by 10,000, followed by lg‐transformation. The top 2000 highly variable genes were identified using “vst” method in Seurat's “FindVariableFeatures” function. The “ScaleData” function was used to scale the normalized data. To group the comparable cells in each dataset, principal component analysis was employed by the “RunPCA” function in Seurat to reduce data dimension using the top variable genes. We performed t‐distributed stochastic neighbor embedding on the top 50 principal components to display high‐dimensional cellular data. The clustering was computed using the KMEANS method. The identity for each cluster was assigned based on prior knowledge of marker genes. Cell trajectory and pseudotime analysis was performed based on the splinefit algorithm in MATLAB. Differentially expressed genes were analyzed using Wilcoxon rank sum test. The t‐SNE plots, UMAP plots, violin plots, bar plots, and heatmaps were generated by scGEAToolbox in MATLAB (Cai, 2020).

### 4.5 Macrophage polarization index (MPI) analysis

Macrophage polarization index was calculated based scRNA‐Seq profiles of signature genes in the polarized bone marrow‐derived macrophages using website: https://macspectrum.uconn.edu (Li et al., 2019).

### 4.6 Liver RNA‐sequencing

Total RNA was isolated from the liver of young, old control, and old AS1842856‐treated mice (*n* = 3 mice/group). The RNA‐Seq was performed at TIGSS molecular genomics core in Texas A&M University. Analysis of RNA ‐seq data was performed at Texas A&M University High Performance Research Computing Institute. Briefly, the STAR aligner program was employed to align RNA sequence reads into mouse genome using STAR. HTseq was used to count the sequences that can be mapped to gene features. DEseq2 was used to normalize the raw read counts and to process differential‐expression gene analysis. The significantly expressed genes were determined by *p*‐value of <0.05.

### 4.7 Flow cytometry

Liver NPCs were washed twice with FACS buffer (PBS, pH 7.4, with 1% BSA and 0.05% Sodium Azide) and incubated with nonspecific IgG to assess background fluorescence. Cells were then incubated with anti‐FcγR II/III antibody (Mouse BD Fc Block; BD Pharmingen) for 15 min on ice, followed by staining with a mixture of fluorescence labeled antibodies including PerCP anti‐CD45 (BD bioscience 557235), Alexa Flour 700 anti‐Ly6G (Biolegend 127622), APC‐cy7 anti‐CD11B (Biolegend 101226), and PE‐cy7 anti‐F4/80 (eBioscience 25–4801‐82) for 30 min on ice. Subsequently, cells were washed and permeabilized for 30 min on ice. For Intracellular markers, PE‐Dazzle594 anti‐TNFα (Biolegend 506345), PE anti‐IL1B antigen (Thermo Fisher Scientific), and APC IL6 antigen (BD Bioscience) were stained for 30 min on ice. Cells were washed before measurement. Flow cytometry was performed using MoFlo Astrios EQ (Beckman Coulter Life Sciences) and data was analyzed using FlowJo software (Tree Star Inc.).

### 4.8 Bone marrow‐derived macrophage isolation

Bone marrow cells were isolated from the tibias and femurs of mice as previously described (Weischenfeldt & Porse, 2008). Cells were seeded into 6‐well plates at a density of 1.5 × 10^6^ cells/well and cultured in a humidified incubator at 37°C and 5% CO_2_ for 7 days. The culture medium was RPMI 1640 medium containing L‐glutamine, 10% fetal bovine serum, 100 U/mL penicillin, and 100 μg/mL streptomycin supplemented with 10 ng/mL macrophage colony‐stimulating factor (M‐CSF). At the end of the 7‐day culture period, >95% of the cells were positive for macrophage markers and bone marrow‐derived macrophages (BMDMs) were subjected to inflammatory assays. On day 7, cells were treated with 5 μM of AS1842856 for 1 h, followed by LPS (100 ng/mL) or saline for 4 h.

### 4.9 Peritoneal macrophage isolation

Peritoneal macrophages (PMs) were obtained from the peritoneum of mice as described (Uysal et al., 1997). Briefly, mice were euthanized by rapid cervical dislocation after anesthetization with isoflurane. Then, 3 mL ice‐cooled phosphate buffered saline (PBS) with 2% fetal bovine serum was injected into the abdominal cavity. After gentle shaking for 3 min, abdominal fluid was collected into tubes using a syringe with an 18G needle. Red blood cells were lysed with ACK lysis buffer for 5 min, and the reaction was stopped by adding two times the volume of PBS. PMs were then collected by centrifugation at 450 *g* for 10 min.

### 4.10 Western blotting

The liver proteins were extracted and adjusted to equal amounts for Western blotting. Foxo1‐S273 phosphorylation antibody was generated as previously described (Wu et al., 2018). Antibodies for Foxo1, pp38‐T180/Y182, p38, pp65, p65, PKA substrates phosphorylation, and GAPDH were purchased from Cell Signaling Technology (Danvers). The protein abundance was quantified using ImageJ (National Institutes of Health).

### 4.11 Serum insulin and triglycerides measurements

Serum insulin and triglycerides levels were measured using mouse insulin ELISA kit (Mercodia) and triglyceride assay kit (Abcam), respectively.

### 4.12 Serum aspartate aminotransferase (AST) and alanine aminotransferase (ALT) activity measurement

Serum AST and ALT activity were determined by commercial AST and ALT activity assay kits (Sigma), respectively.

### 4.13 Serum lipid profile analysis

Serum total cholesterol, NEFA, HDL, LDL, and ALP were measured using DxC 700 AU Chemistry Analyzer (Beckman Coulter).

### 4.14 H&E staining

Liver samples were fixed in 4% formaldehyde. After dehydration, liver samples were embedded into paraffin and then 5 μm liver slides were prepared. Liver slides were stained with hematoxylin after hydration with gradient alcohol, followed by eosin staining. Finally, the slides were dehydrated and sealed. The liver slides were observed using Aperio Slide Scanner (Leica Biosystems Inc.).

### 4.15 Statistical analysis

All results are presented as mean ± SEM. *p* values were calculated using Student's *t* test for the comparison of differences between two groups. *p* < 0.05 was considered statistically significant.

## AUTHOR CONTRIBUTIONS

Shaodong Guo and Wanbao Yang were involved in conceptualization. Wanbao Yang, Da Mi Kim, Wen Jiang, Weiqi Ai, Quan Pan, Shahina Rahman, James J. Cai, and Wesley A. Brashear were involved in methodology. Wanbao Yang and Da Mi Kim were involved in investigation and writing—original draft preparation. Wanbao Yang, Da Mi Kim, Wen Jiang, Weiqi Ai, and Quan Pan were involved in data acquisition. Shaodong Guo and Yuxiang Sun were involved in writing—review and editing. Shaodong Guo was involved in supervision.

## CONFLICT OF INTEREST STATEMENT

The authors declare no conflict of interest.

## Supporting information


Data S1

## Data Availability

All the data presented here are available from the corresponding authors upon reasonable request.
