# Supplementary material for: Suppression of FOXO1 attenuates inflamm‐aging and improves liver function during aging
Source: Aging Cell. 2023 Aug 21;22(10):e13968. doi: 10.1111/acel.13968 (PMC10577549; doi:10.1111/acel.13968)
Supplement: Supplementary file 1 — Data S1 [file ACEL-22-e13968-s001.pdf]

Suppression of FOXO1 attenuates inflamm-aging and improves liver function during aging

Supplementary Materials

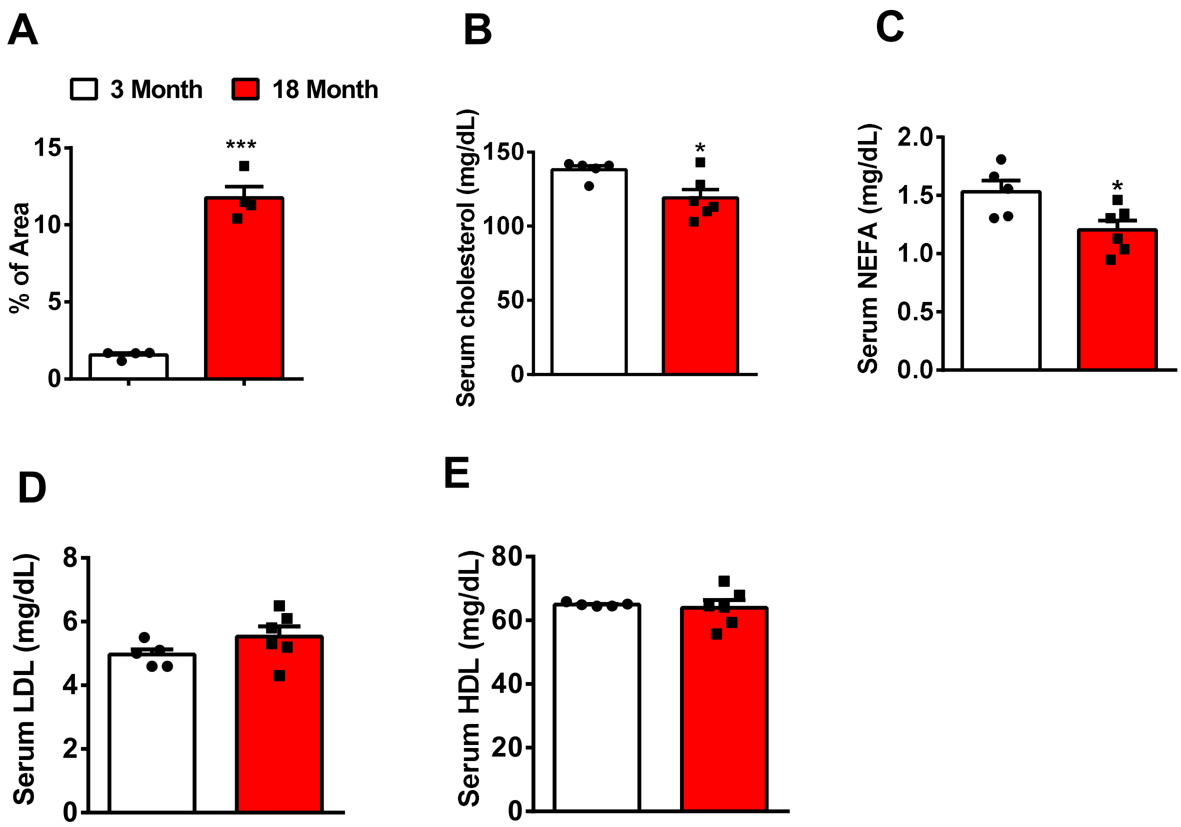

Supplementary Fig. 1 Lipid and glucose homeostasis are impaired in old mice
(A) Percentage of fat area in the livers of young and old mice, n=4 mice/group. (B-E) Serum cholesterol (B), NEFA (C), LDL (D), and HDL (E) in young and old mice, n=5-6 mice/group. All data are presented as mean ± SEM. * $p < 0.05$.

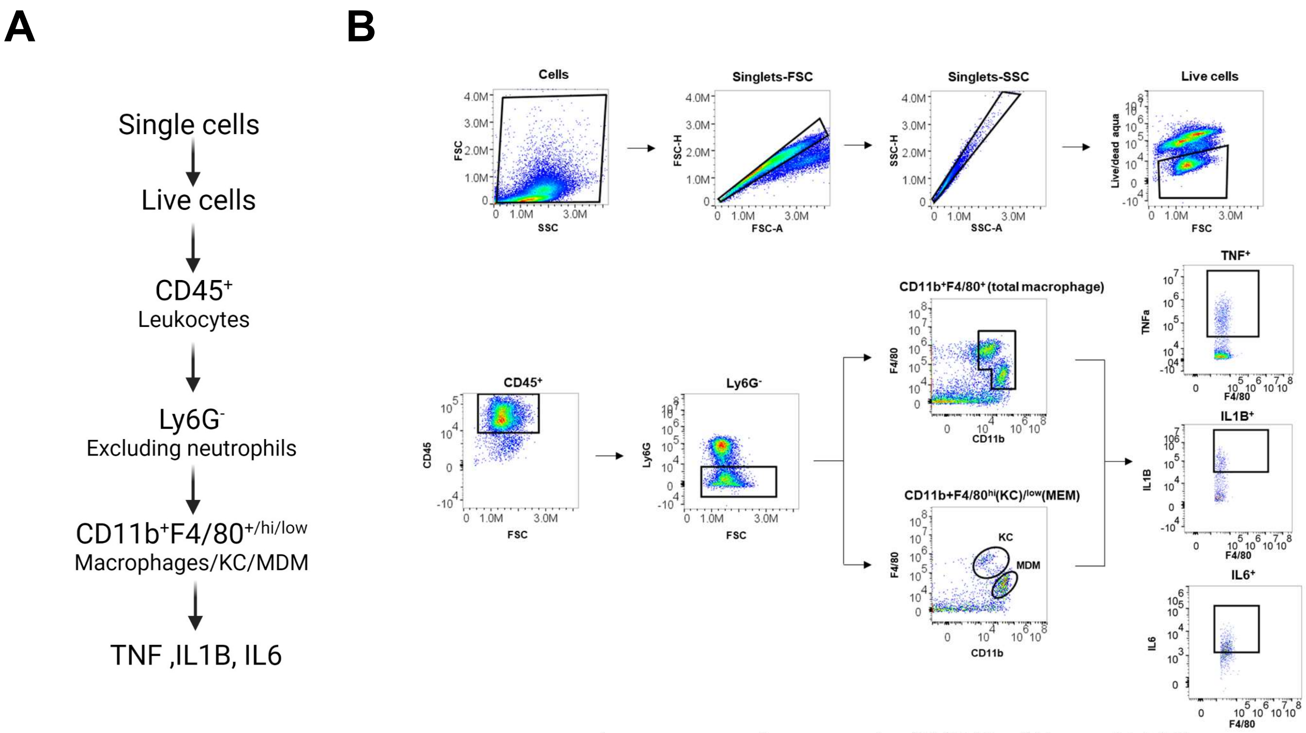

Supplementary Fig. 2 . Flow cytometry gating strategy in control of NPC of liver. (A) Flow cytometry gating strategy of liver NPCs. (B) Representative scatter-blots of flow cytometry analysis.

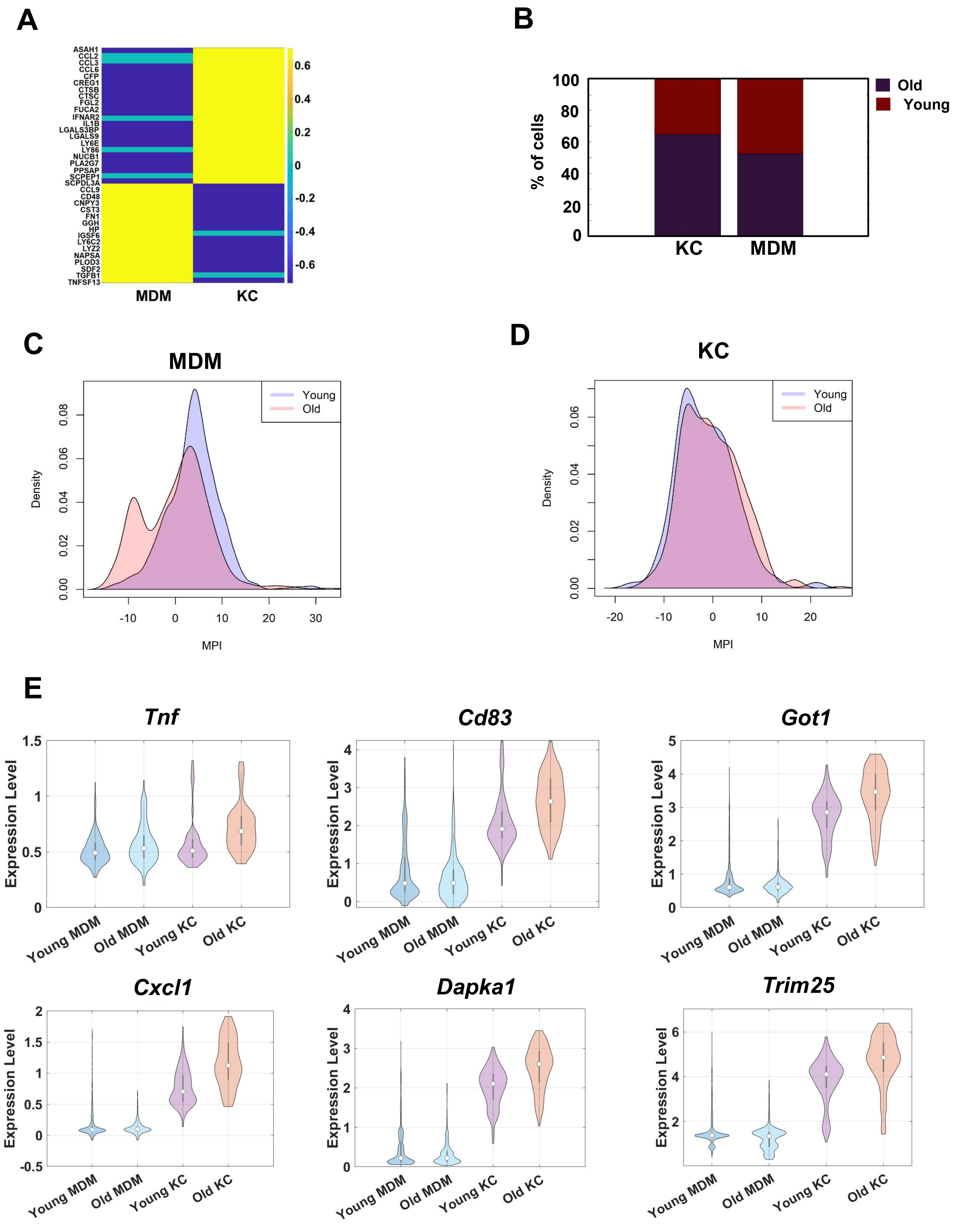

A
ASAH1
CCL2
CCL3
CCL6
CFP
CREG1
CTSB
CTSC
FGL2
FUCA2
IFNAR2
IL1B
LGALS3BP
LGALS9
LY6E
LY86
NUCB1
PLA2G7
PPSAP
SCPEP1
SCPDL3A
CCL9
CD48
CNPY3
CST3
FN1
GGH
HP
IGSF6
LY6C2
LYZ2
NAPSA
PLOD3
SDF2
TGFB1
TNFSF13
MDM
KC
0.6
0.4
0.2
0
-0.2
-0.4
-0.6
B
% of cells
Old
Young
KC
MDM
C
MDM
Density
MPI
Young
Old
D
KC
Density
MPI
Young
Old
E
Tnf
Cd83
Got1
Cxcl1
Dapka1
Trim25
Expression Level
Young MDM
Old MDM
Young KC
Old KC

Supplementary Fig. 3 Kupffer cells contribute to the pro-inflammation in hepatic macrophages during aging.

(A) Heatmap of macrophage-secreted factor gene expression in KCs and MDMs. (B) Percent contribution of young and old mice in KCs and MDMs. (C) Macrophage polarization index (MPI) analysis in the KCs of young and old mice. (D) Macrophage polarization index (MPI) analysis in the MDMs of young and old mice. (E) Violin plots of ARKC genes in KCs and MDMs of young and old mice.

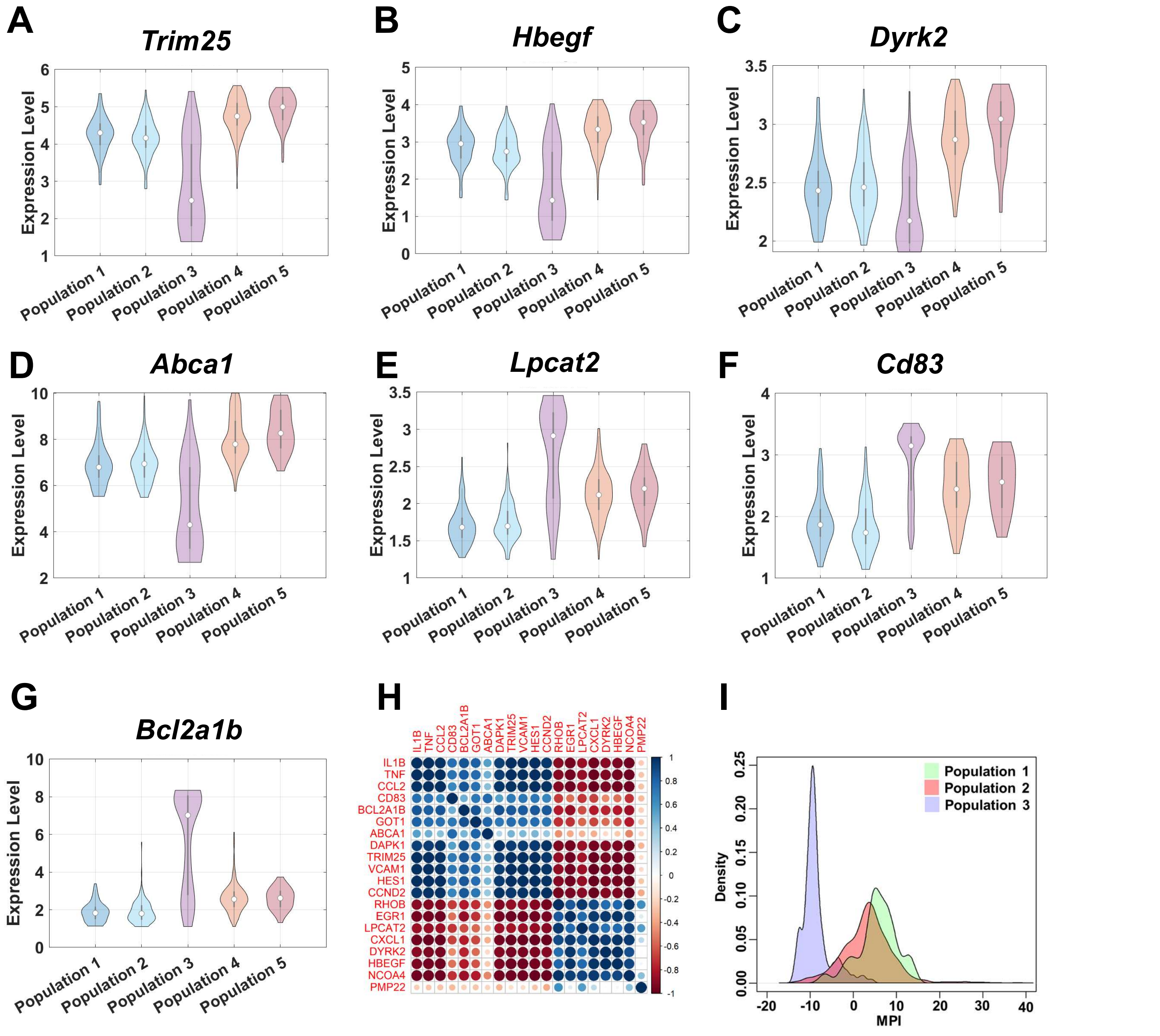

Supplementary Fig. 4 . KCs and MDMs are differentially affected during aging. inhibition improves aging-induced inflammation in macrophages.

(A-G) Violin plots of ARKC gene expression in KC populations. (H) Correlation between ARKC gene expression and pro-inflammatory cytokine gene expression in KC population 3. (I) Macrophage polarization index (MPI) analysis in MDM populations.

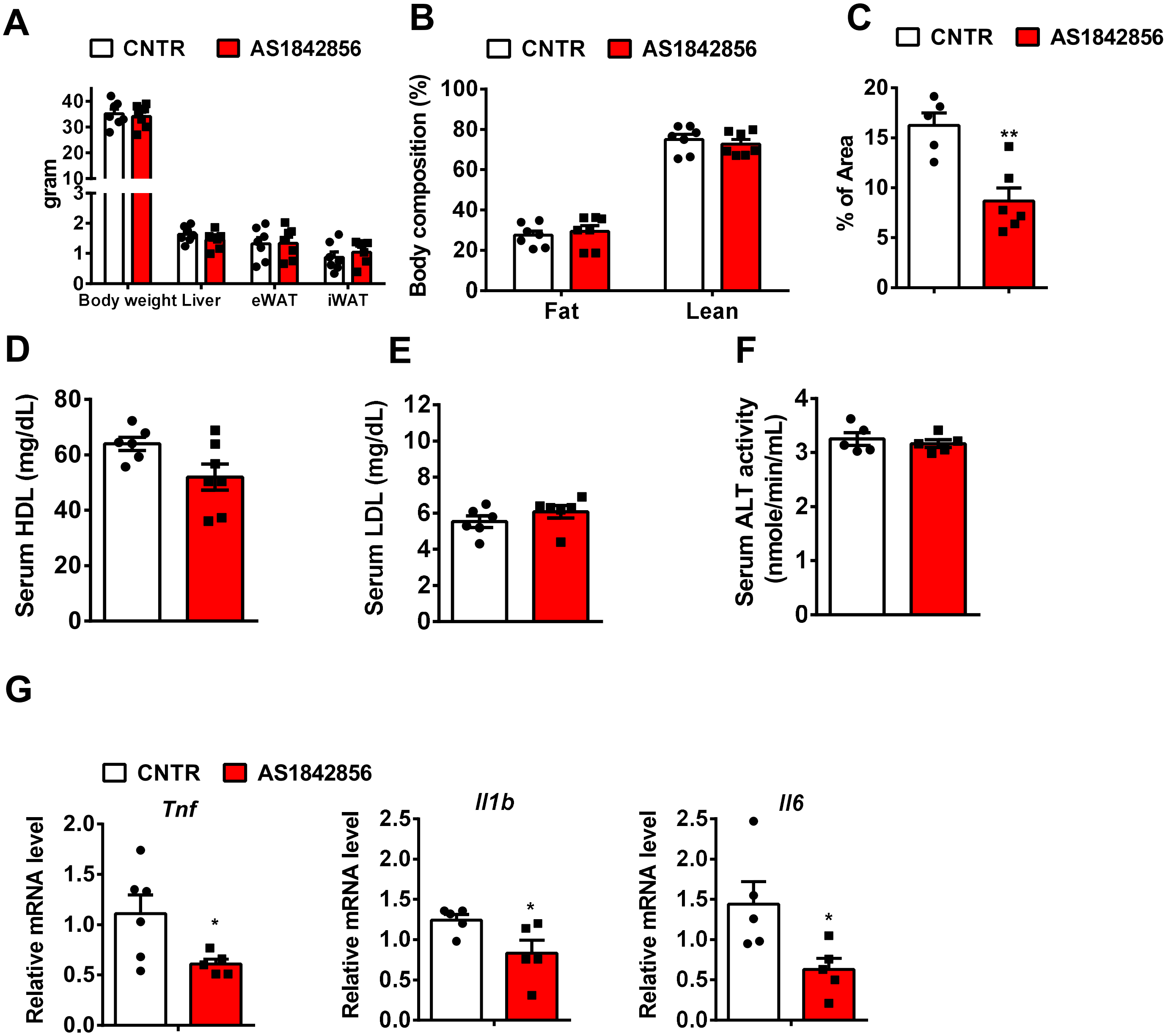

Supplementary Fig. 5 FOXO1 inhibition improves glucose homeostasis, hepatic steatosis, and chronic inflammation in old mice.

(A) Body weight and tissue weight in control and AS1842856-treated old mice, n=7. (B) Body composition in control and AS1842856-treated old mice, n=7. (C) Percentage of fat area in the livers of control and AS1842856-treated old mice, n=5-6 mice/group. (D-F) Serum HDL, LDL, and ALT in control and AS1842856-treated old mice, n=5-7. (G) The mRNA expression of pro-inflammatory cytokine genes in the PMs of control and AS1842856 treated old mice, n=5-6. All data are presented as mean ± SEM. * $p < 0.05$, ** $p<0.01$.

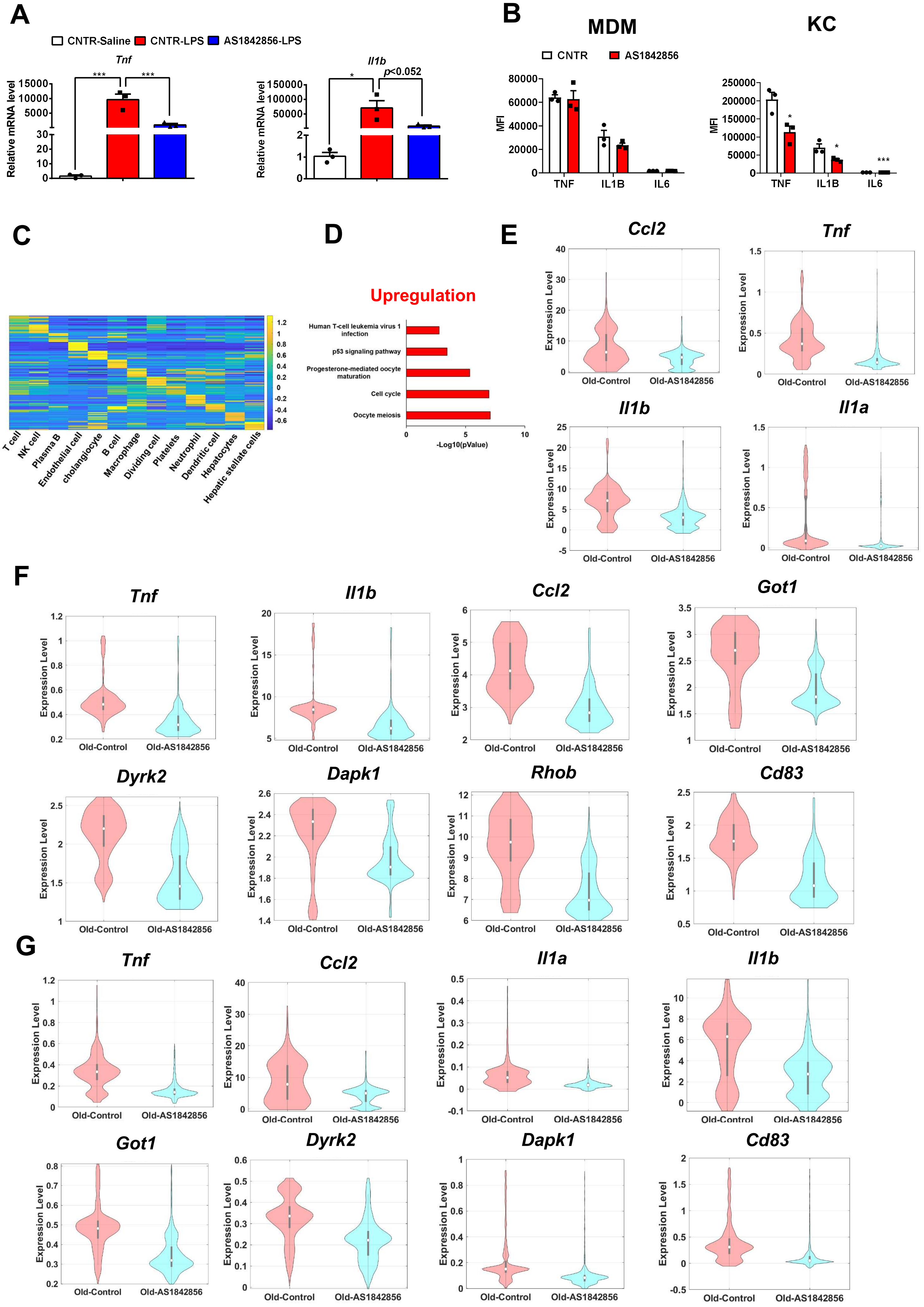
A
CNTR-Saline
CNTR-LPS
AS1842856-LPS
Tnf
Il1b
Relative mRNA level
p<0.052
B
MDM
KC
CNTR
AS1842856
MFI
TNF
IL1B
IL6
C
T cell
NK cell
Plasma B
Endothelial cell
cholangiocyte
B cell
Macrophage
Dividing cell
Platelets
Neutrophil
Dendritic cell
Hepatocytes
Hepatic stellate cells
D
Upregulation
Human T-cell leukemia virus 1 infection
p53 signaling pathway
Progesterone-mediated oocyte maturation
Cell cycle
Oocyte meiosis
-Log10(pValue)
E
Ccl2
Tnf
Il1b
Il1a
Expression Level
Old-Control
Old-AS1842856
F
Tnf
Il1b
Ccl2
Got1
Dyrk2
Dapk1
Rhob
Cd83
G
Tnf
Ccl2
Il1a
Il1b
Got1
Dyrk2
Dapk1
Cd83

Supplementary Fig. 6 FOXO1 inhibition improves pro-inflammation in hepatic macrophages during aging. (A) The mRNA expression of *Tnf* and *Il1b* in BMDMs treated with AS1842856, n=3. (B) The MFI of pro-inflammatory cytokines in the MDMs and KCs of control and AS1842856-treated old mice, n=3 mice/group. (C) Heatmap of cluster marker genes in the liver cells of control and AS1842856-treated old mice. (D) Pathway analysis of upregulated genes in the hepatic macrophages of AS1842856-treated old mice. (E) Violin plots of chemokine and pro-inflammatory cytokine genes in the hepatic macrophages of control and AS1842856-treated old mice. (F) Violin plots of pro-inflammatory cytokine and ARKC gene in the KCs of control and AS1842856-treated old mice. (G) Violin plots of pro-inflammatory cytokine and ARKC gene in the MDMs of control and AS1842856-treated old mice. All data are presented as mean ± SEM. * $p < 0.05$, *** $p < 0.001$.

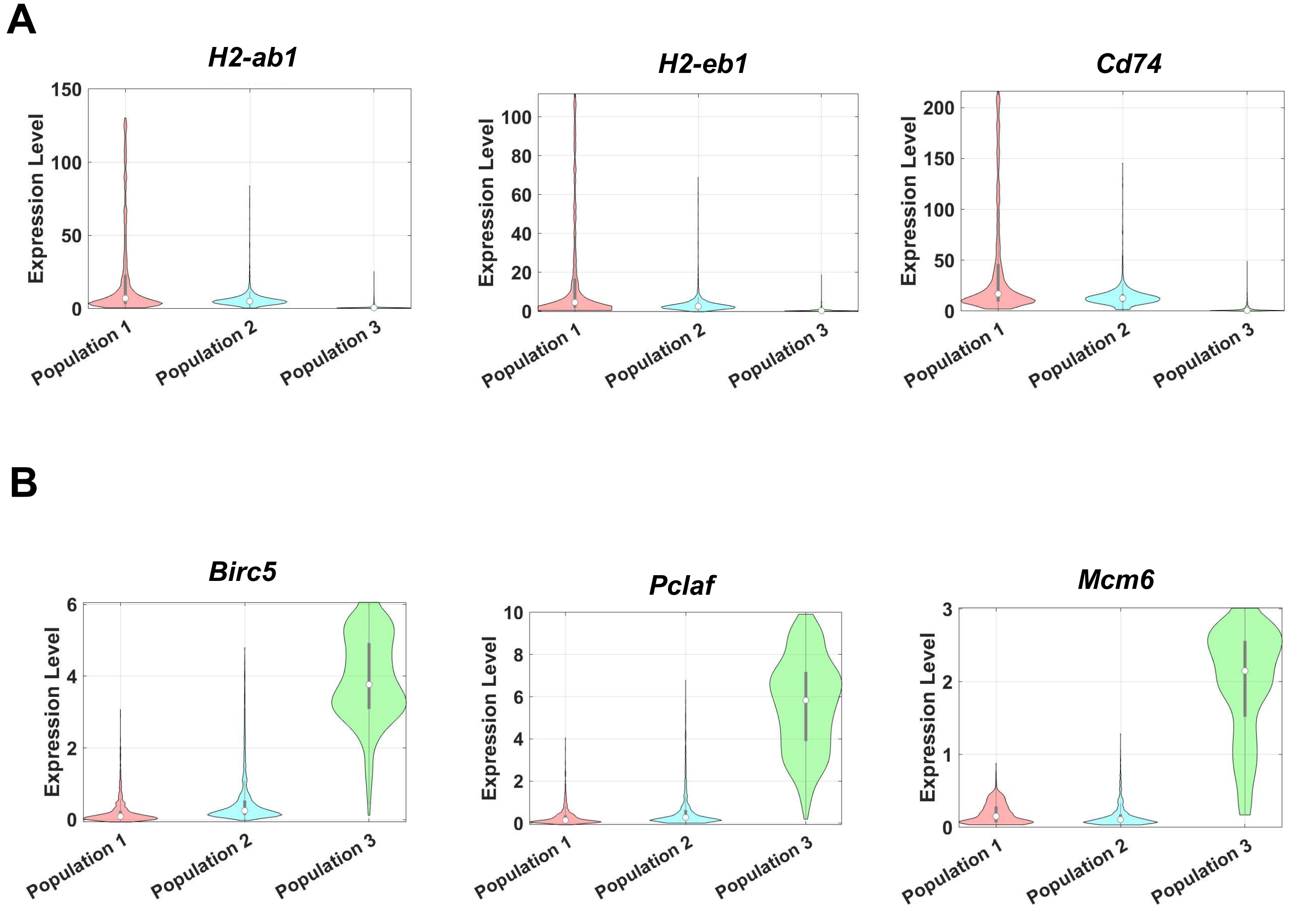

Supplementary Fig. 7 FOXO1 inhibition rescues aging-induced phenotypes in KC but not in MDM.

(A) Violin plots of antigen presentation/process gene expression in MDM populations. (B) Violin plots of aging-induced MDM marker gene expression.
